# Tumor-derived vesicles in immune modulation: focus on signaling pathways

**DOI:** 10.3389/fimmu.2025.1581964

**Published:** 2025-05-15

**Authors:** Fei Yin, Yangfang He, Yue Qiao, Yan Yan

**Affiliations:** ^1^ Department of Neurology, The Second Hospital of Jilin University, Changchun, China; ^2^ Department of Endocrinology and Metabolism, The Second Hospital of Jilin University, Changchun, China; ^3^ Department of Physical Examination Center, The Second Hospital of Jilin University, Changchun, China; ^4^ Department of Endocrinology, The Second Hospital of Jilin University, Changchun, China

**Keywords:** TdEVs, immune modulation, signaling pathways, immunotherapy, tumor

## Abstract

Tumor-derived extracellular vesicles (TDEVs) represent a heterogeneous population of extracellular vesicles (EVs), including exosomes, microvesicles, and apoptotic bodies, which are essential for tumor growth. EVs function as natural carriers of bioactive molecules, including lipids, proteins, and nucleic acids, enabling them to influence and regulate complex cellular interactions within the tumor microenvironment (TME). The TDEVs mainly have immunosuppressive functions as a result of the inhibitory signals disrupting the immune cell anti-tumor activity. They enhance tumor progression and immune evasion by inhibiting the effector function of immune cells and by altering critical processes of immune cell recruitment, polarization, and functional suppression by different signaling pathways. In this sense, TDEVs modulate the NF-κB pathway, promoting inflammation and inducing immune evasion. The Janus kinase (JAK)-signal transducer and activator of transcription (STAT) signaling is required for TDEV-mediated immune suppression and the manifestation of tumor-supporting features. The phosphoinositide 3-kinase (PI3K)/protein kinase B (Akt)/mammalian target of rapamycin (mTOR) signaling, necessary for metabolic reprogramming, is orchestrated by TDEV to abrogate immune response and drive cancer cell proliferation. Finally, exosomal cargo can modulate the NOD-, LRR-, and pyrin domain-containing protein 3 (NLRP3) inflammasome, activating pro-inflammatory responses that influence tumor development and immunomodulation. In this review, we take a deep dive into how TDEVs affect the immune cells by altering key signaling pathways. We also examine emerging therapeutic approaches aimed at disrupting EV-mediated pathways, offering promising avenues for the development of novel EV-based cancer immunotherapy.

## 1 Introduction

Tumor-derived extracellular vesicles (TDEVs) play a significant role in the tumor microenvironment (TME), impacting tumor progression, immune responses, and systemic physiology (1). These vesicles play a pivotal role in cell-to-cell communication by enabling continuous interactions between tumor cells and various elements of the immune system (2). TDEVs stimulate the secretion of tumor necrosis factor alpha (TNF-α) and Interleukin (IL)-1 beta (IL-1β) from CD14^+^ monocytes, leading to nuclear factor kappa B (NF-κB) activation (3). Popěna et al. (4) found that extracellular vesicles (EVs) secreted from colorectal cancer (CRC) modulate the immunophenotype of monocytes and macrophages, resulting in an increase in CD14^+^ in M0 and human leukocyte antigen-DR (HLA-DR) in M1 (pro-inflammatory) and M2 (anti-inflammatoryM2) macrophages. Another study found that EVs released from CRC cells containing high levels of miR-21-5p and miR-200a promote immune escape by enhancing the expression of programmed death (PD)-ligand 1 (PD-L1) in tumor-associated macrophages (TAMs), correlating with a poorer prognosis (5). EVs derived from glioblastoma that contain PD-L1 promote the development of immunosuppressive monocytes, which subsequently inhibit T-cell proliferation (6). Breast cancer cells, in response to endoplasmic reticulum (ER) stress, release exosomes enriched with miR-27a-3p, which are transferred to macrophages and facilitate tumor immune evasion by enhancing PD-L1 expression through the phosphatase and tensin homolog (PTEN)/phosphoinositide 3-kinase (PI3K)/protein kinase B (Akt) pathway (7).

Macrophage polarization is affected by significant intracellular metabolic alterations (8). Generally, M1 macrophages sustain their pro-inflammatory functions through aerobic glycolysis, whereas M2 macrophages depend primarily on oxidative phosphorylation (OXPHOS) to support their anti-inflammatory activities (9). In this regard, hypoxic TDEV-pyruvate kinase M2 (PKM2) has been shown to induce macrophage polarization toward the M2 phenotype via activation of Adenosine monophosphate (AMP)-activated protein kinase (AMPK) pathway, thereby promoting lung cancer progression and metastasis (10). TDEVs enhance macrophage glycolysis via the Toll-like receptor (TLR) 2-NF-κB, promoting an immunosuppressive phenotype characterized by high PD-L1 expression (11). PD-L1 upregulation mediated by melanoma-derived EVs involves TLR signaling via the Heat shock protein (HSP)86/TLR4 axis (12). The uptake of EVs by macrophages activates TLR2 and TLR4, leading to cytokine production (13, 14). Breast cancer-derived exosomes carrying miR-9 and miR-181a contribute to the expansion of myeloid-derived suppressor cells (MDSCs) by downregulating suppressor of cytokine signaling 3 (SOCS3) and protein inhibitor of activated signal transducer and activator of transcription 3 (STAT3) (PIAS3), respectively (15). This suppression leads to elevated IL-6 expression and hyperactivation of the JAK/STAT, primarily due to the loss of SOCS3-mediated regulation (15). Momen-Heravi et al. (16) showed that EVs released from head and neck squamous cell carcinoma (HNSCC) are internalized by monocytes, triggering activation of the NF-κB, which in turn enhances the production of matrix metalloproteinase (MMP) 9 and enhances cyclooxygenase-2 (COX-2)expression levels, prostaglandin E2 (PGE2), and vascular endothelial growth factor (VEGF) after stimulation of THP-1 cells. This review provides an in-depth overview of how TDEVs alter immune cell function via key signaling pathways, including NF-κB, PI3K/Akt/mammalian target of rapamycin (mTOR), JAK/STAT, and transforming growth factor-β (TGF-β). Additionally, we present novel therapies that target EV-mediated processes to provide further potential TDEV-based cancer immunotherapeutics.

## 2 Tumor-derived vesicle characterization and biogenesis

**Figure 1:**
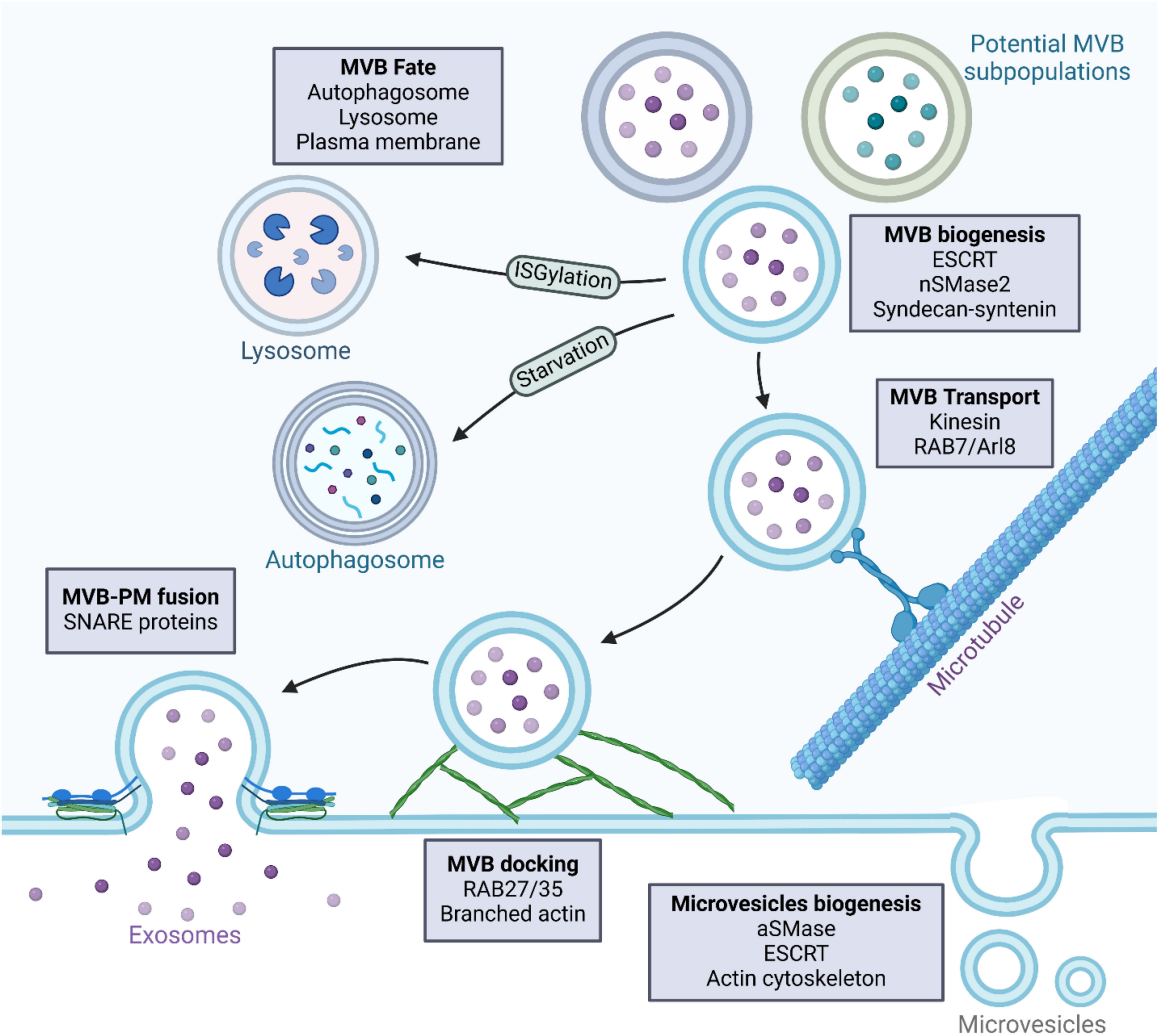
Biogenesis of tumor-derived vesicles. Tumor-derived vesicles are mainly composed of microvesicles and exosomes, which are different in their biogenesis and molecular composition. Microvesicles are formed by direct outward budding from the plasma membrane. This is initiated by plasma membrane remodeling, in which localized changes in lipid composition, along with cytoskeletal reorganization, cause membrane protrusions. Lipid raft domains and alterations in phospholipid asymmetry contribute to this process. Cytoskeletal rearrangements, mechanistically in large part mediated by actin and myosin-driven contractile forces, enable the pinching off of microvesicles from the plasma membrane. Key regulators include the Rho GTPases, ARF6, and the Endosomal Sorting Complex Required for Transport (ESCRT). Once formed, microvesicles bud and are released into the extracellular space, carrying molecular cargo that reflects the characteristics of the tumor cell of origin. In contrast, exosomes are derived from the endosomal pathway, specifically from multivesicular bodies (MVBs) containing intraluminal vesicles (ILVs). Their formation begins with the maturation of early endosomes into late endosomes, which is followed by the production of MVBs. The ESCRT machinery or lipid-mediated mechanisms, such as ceramide accumulation, drive the formation of ILVs within MVBs. Once formed, MVBs are subjected to several possible fates. They may fuse with the plasma membrane to release exosomes into the extracellular space, be directed to lysosomes for degradation, or interact with autophagosomes, although in most cases the mechanisms defining this fate are not well understood. MVBs that are destined for the secretion of exosomes are transported along microtubules toward the plasma membrane under the control of Rab GTPases like Rab11, Rab27a/b, and Rab35. Docking at the plasma membrane is a prerequisite for exosome release and is mediated by SNARE proteins that facilitate membrane fusion.

EVs comprise a diverse collection of membrane-bound vesicles essential for various physiological and pathological processes (17). They carry molecules such as proteins, lipids, and nucleic acids, and are associated with diverse functions (18). Exosomes and ectosomes are types of EVs secreted by normal and tumor cells into the extracellular microenvironment (19). TDEVs influence tumor development and metastasis by modulating immune, endothelial, and epithelial cells (18). They may contribute to tumor progression by enhancing tumor aggressiveness, invasiveness, angiogenesis, extracellular matrix remodeling, drug resistance, and immunosuppression (20, 21). The delivery of metastasis-associated components, such as proteins or microRNAs (miRNAs), can initiate and alter signaling pathways, thereby modifying the behavior and functions of target cells (22). EVs are categorized into three types according to their biogenesis pathway: exosomes, ectosomes, and apoptotic bodies (23). Exosomes are small vesicles, typically under 150 nanometers in size, formed when multivesicular bodies (MVBs) merge with the cell membrane and release their contents (24). Lastly, ectosomes, also referred to as microvesicles, are generated through outward budding from the plasma membrane and typically measure between 100 and 1,000 nanometers in diameter (25). The tetraspanin protein family is recognized as a crucial cellular effector in the biogenesis of vesicles, with distinct functions in exosome and ectosome formation (26). Apoptotic bodies are membrane-bound vesicles that are generated as a result of programmed cell death (apoptosis), encapsulating cellular components for clearance by phagocytes (27). The secretion of EVs into the extracellular matrix occurs as a result of the fusion between the outer membrane of MVBs with the plasma membrane of the originating cells, a process initiated by invagination of the plasma membrane during cell maturation, giving rise to endosomes, which are further categorized into early endosomes—mostly peripheral to the cell—and late endosomes—proximal to the nucleus (28, 29). Eventually, late lysosomes undergo a fusion process with the plasma membrane, resulting in the degradation of MVBs and the subsequent release of EVs into the extracellular environment (30). **Figure 1** provides an overview of TDEV biogenesis.

## 3 Tumor-derived vesicles and immune modulation

Emerging evidence indicates that EVs have a function in modulating immune responses, functioning either as immune suppressors or stimulators, depending on their intricate molecular composition (**Table 1**) (47). EVs derived from cancer cells assist in immune system evasion by strengthening the tumor’s ability to withstand immune-mediated responses (48). This effect depends on the specific immunosuppressive molecules present on the EVs and their interaction with related receptors on target immune cells (49). TDEVs contain not only immunosuppressive molecules but also costimulatory molecules, including major histocompatibility complex (MHC) I and II, growth-promoting cytokines, and various tumor-associated antigens (TAAs) (47).

**Table 1:** Overview of tumor-derived vesicles in modulation of immune cells.

Vesicle	Study	Tumor	Immune cell	Function	Ref.
Exosome	*In vitro*	Melanoma	Macrophage	Exosomes derived from melanoma induced a hybrid activation profile in macrophages, showing characteristics of both pro-tumorigenic M1 and M2 phenotypes.	(31)
Exosome	*In vitro*	Osteosarcoma	Macrophage	Osteosarcoma exosomes promoted M2 polarization, which facilitated tumor invasion and metastasis.	(32)
Extracellular vesicle (EV)	*In vivo*	Ovarian cancer	Macrophage	Tumor-EVs contribute to create an immunosuppressive environment that favors ovarian cancer progression and proliferation.	(33)
EV	*In vitro* and *in vivo*	Ovarian cancer	Macrophage	EVs from ovarian cancer-induced M2 macrophage polarization, which promoted ovarian cancer progression.	(34)
EV	*In vitro* and *in vivo*	Colorectal cancer	Macrophage	Colorectal cancer-derived EVs promoted the macrophage polarization of toward M2 by inhibiting histone deacetylase 11 (HDAC11) expression.	(35)
Exosome	*In vitro* and *in vivo*	Breast cancer	Dendritic cell (DC)	Tumor-derived exosomes inhibited the development and migration of DCs but promoted their immunosuppressive activity.	(36)
Exosome	*In vivo*	Breast cancer	DC	Tumor-derived exosomes suppress the differentiation of human DCs.	(37)
Microvesicle	Clinical	Acute myeloid leukemia	Natural killer (NK) cell	Acute myeloid leukemia-derived microvesicles suppressed NK cell cytotoxicity and activity.	(38)
EVs	*In vitro* and *in vivo*	Pancreatic Cancer	NK	Exosomes from pancreatic cancer cells inhibit NK cell function, which can be one mechanism by which metastatic tumor cells evade immune detection in the pre-metastatic niche.	(39)
Small EVs	*In vivo*	Ovarian cancer	CD4^+^ and CD8^+^ T-cells	Small EVs isolated from ovarian cancer suppressed T cell proliferation.	(40)
Exosome	*In vitro*	Head and neck cancer	CD8^+^ T cell	Tumor-EVs secretion enhanced the immunosuppressive features of tumors by modifying CD8^+^ T lymphocyte function in the TME.	(41)
Exosome	Clinical and *in vitro*	Neck squamous cell carcinoma	T-lymphocyte	A difference in gene expression changes mediated by exosomes from resting and activated T cells was found. This underlined that TDEVs are differentially important for modulating gene expression and T-cell functions in CD4^+^ conventional T cells compared with Treg cells.	(42)
EVs	*In vitro*	Melanoma and breast cancer	T cell	EVs released by tumor cells can suppress T-cell growth and promote their aging, highlighting a unique mechanism through which tumors contribute to immune suppression within TME.	(43)
Exosome	*In vivo*	Mammary adenocarcinomas	Myeloid-derived suppressor cell (MDSC)	Elements in the TME facilitated the exosome-mediated expansion of MDSCs in the tumor.	(44)
EV	*In vivo*	Acute myeloid leukemia	MDSC	Acute myeloid leukemia-derived EVs were taken up by myeloid progenitor cells, thus facilitating the preferential expansion of MDSCs cells over functionally competent antigen-presenting cells (APCs).	(45)
EV	*In vitro*	Gastric cancer	Neutrophil	Exosomes derived from the gastric cancer microenvironment induce neutrophils to inhibit T-cell immunity.	(46)

Indeed, many studies have suggested that TDEVs convey suppressive or activating molecular signals, which reprogram tumor-infiltrating immune cells (TIICs) to adopt crucial roles in tumor progression by manifesting immunosuppressive functions through the recruitment and differentiation of regulatory B (Breg) cells, regulatory T (Treg) cells, TAMs, MDSCs, and neutrophils (50). Hepatocellular carcinoma (HCC)-secreted exosomes contain high mobility group box 1 (HMGB1), which is responsible for the promotion of T-cell immunoglobulin and mucin domain 1 (TIM-1)^+^ Breg cells (51). These Breg cells help establish an immunosuppressive environment by producing IL-10 and suppressing the activity of CD8 T cells (51). Tumor-derived exosomal TGF-β promotes the differentiation of Treg cells from peripheral blood precursors, and these expanded Tregs subsequently secrete TGF-β to maintain their immunosuppressive function (52). One such mechanism involves tumor-derived exosomal surface HSP72 that, through the HSP72-TLR axis, initiates the signaling for the T cell-dependent immunosuppressive function of MDSCs, ultimately leading to STAT3 activation (53). Additionally, exosomes derived from tumors can drive monocyte differentiation into MDSC via TGF-β signaling, thereby inhibiting T cell proliferation and cytotoxic activity (54). The transfer of PKM2 from HCC-derived ectosomes to monocytes promotes their differentiation into M2-like TAMs (55). These macrophages contribute to HCC progression by releasing factors that support tumor growth through a process resembling regurgitation feeding (55). TDEVs play a significant role in tumor immune evasion and progression through the regulation and reprogramming of TIICs (56, 57). These vesicles influence the TME through various mechanisms, including the recruitment and promotion of immunosuppressive cell differentiation, such as Tregs, Bregs, TAMs, and neutrophils, thereby intensifying immune suppression within the TME (55, 58). TDEVs promote immune evasion by transferring immunosuppressive factors, including PD-L1 and Fas ligand, to immune cells, thereby inhibiting their anti-tumor functions (59, 60). Also, EVs produced by tumors can suppress the anti-tumor immune response in distant tissues, fostering an immunosuppressive microenvironment conducive to metastatic niche formation (61, 62). In summary, the findings show that TDEVs play a significant role in immune modulation depending on their molecular composition.

## 4 Tumor-derived vesicles and various aspects of immune modulation with a focus on the signaling pathways

**Figure 2:**
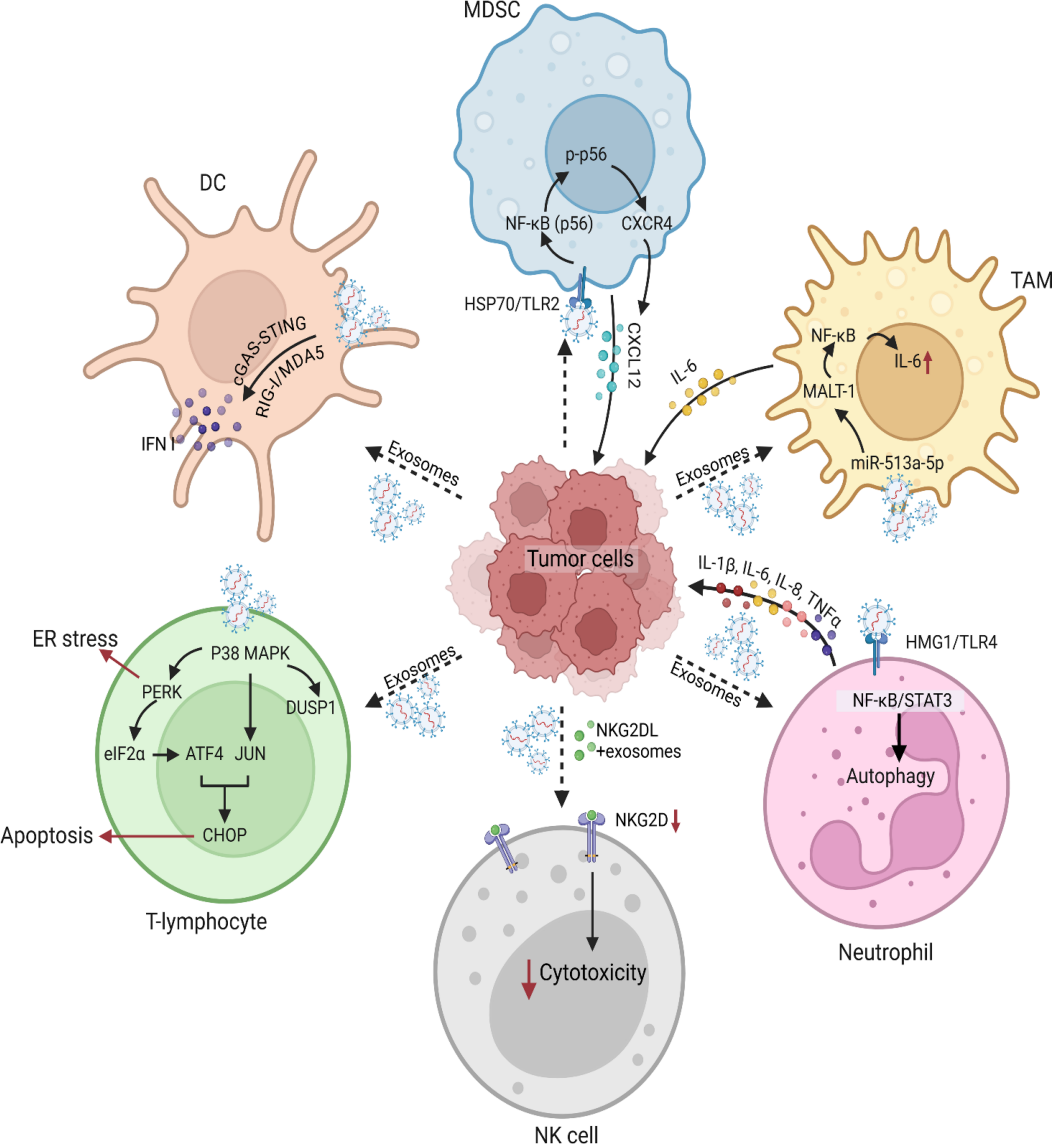
Tumor-derived vesicles and induced signaling modulation in various immune cells. Tumor-derived vesicles play a critical role in modulating immune signaling pathways. These vesicles carry a diverse array of molecules such as DNA, RNA, proteins, and lipids, which interact with various immune cell populations to alter their function and phenotype. Tumor-derived exosomes also exert immunosuppressive effects by interfering with immune responses and cytotoxic functions of immune cells. For instance, tumor cells secrete exosomes enriched with epidermal growth factor receptor (EGFR), which are taken up by macrophages. These EGFR+ exosomes modulate innate immune responses by deregulating interferon regulatory factor 3 (IRF3) through mitogen-activated protein kinase kinase 2 (MEKK2), thereby dampening immunity. Furthermore, tumor-derived microvesicles and exosomes carrying ligands for the NK group 2 member D (NKG2D) receptor downregulate NKG2D expression on DCs, impairing their cytotoxic activity. This suppression of immune effector functions contributes to immune escape and enhances tumor survival within the microenvironment. One of the key mechanisms by which tumor-derived vesicles impact immune function involves their interaction with DCs. Cancer cells release tumor-derived exosomes carrying double-stranded DNA (dsDNA), which are internalized by DCs, leading to their activation and the release of type I IFN. These findings highlight the role of tumor-derived vesicles in shaping DC-mediated immune responses, which can either enhance antitumor immunity or facilitate immune evasion. Another significant aspect of tumor-derived exosomes is their role in the recruitment and expansion of MDSCs, which are key players in tumor-induced immunosuppression. Prostate cancer-derived exosomes have been shown to promote the migration of MDSCs into the tumor microenvironment by upregulating CXCR4 via the TLR2/NF-κB signaling pathway. MDSCs exhibited enhanced migration toward exosome-enriched environments, an effect that was mitigated by the CXCR4-specific inhibitor AMD3100. Analyses further confirmed a marked increase in CXCR4 expression in MDSCs upon exosome stimulation, along with elevated TLR2 and NF-κB activation. Blocking TLR2 with a specific inhibitor (C29) significantly reduced the expression of phosphorylated p65 (p-p65) and CXCR4, reinforcing the notion that TLR2-mediated NF-κB activation is a key driver of MDSC recruitment via the CXCR4-CXCL12 axis. In addition to their effects on innate immune cells, tumor-derived exosomes have been implicated in modulating T lymphocyte function. Prostate cancer-derived exosomes have been shown to integrate with T lymphocytes, leading to the activation of the p38 mitogen-activated protein kinase (MAPK) pathway. Phosphorylated p38 MAPK subsequently triggers endoplasmic reticulum (ER) stress, activating the PERK-eIF2α-ATF4-CHOP signaling axis, which culminates in T cell apoptosis. Interestingly, the dual-specificity phosphatase 1 (DUSP1) was upregulated following p38 MAPK activation, suggesting a potential negative feedback mechanism to regulate MAPK signaling. Pharmacological inhibition of p38 MAPK using SB203580 attenuated ER stress, thereby protecting T lymphocytes from apoptosis. Further investigations into tumor-derived vesicle-mediated immune modulation have uncovered a link between IL-6 and the activation of the JAK2-STAT3 signaling pathway in tumor cells. Specifically, exosomes from TAMs were found to promote the expression of eukaryotic initiation factor 4A3 (EIF4A3) in breast cancer cells through JAK2-STAT3 activation. Gene set enrichment analysis (GSEA) confirmed the significant upregulation of genes associated with the JAK2-STAT3 pathway in breast cancer cells co-cultured with TAM-derived exosomes. Chromatin immunoprecipitation (ChIP) and luciferase reporter assays validated the direct interaction between STAT3 and the EIF4A3 promoter. In the context of neutrophil, gastric cancer-derived exosomes have been shown to prolong neutrophil survival and promote the expression of inflammatory factors, further enhancing gastric cancer cell migration. Gastric cancer-derived exosome -mediated neutrophil activation is primarily driven by the high mobility group box-1 (HMGB1) protein, which interacts with TLR4 to activate the NF-κB pathway. This interaction triggers an autophagic response in neutrophils, reinforcing their pro-tumor activity. Blocking HMGB1/TLR4 interactions, inhibiting the NF-κB pathway, or suppressing autophagy reversed gastric cancer-derived exosome-induced neutrophil activation. These findings suggest that gastric cancer-derived exosomes facilitate a tumor-supportive neutrophil phenotype through HMGB1/TLR4/NF-κB signaling, shedding light on the complex interplay between exosomes and immune cells in shaping the tumor microenvironment.

TDEVs are crucial modulators of TME and also control many aspects of immunity (63). These vesicles have been regulating the immune responses through different signaling pathways (**Figure 2**). This section provides an in-depth view of the signaling pathways with an emphasis on the mechanism of action and the function of the TDEVs in the modulation of immune cells (**Table 2**).

**Table 2:** Summary of tumor-derived vesicles and induced signaling modulation in immune cells.

Tumor-derived vesicle	Study	Immune cell	Signaling	Role	Description	Ref.
Melanoma-derived microvesicle	Clinical and *in vitro*	Monocyte	TGF-β	Dendritic cell (DC) differentiation	Microvesicles from stage IV melanoma patients had similar effects on CD14^+^ monocytes, directing their differentiation into CD14^+^HLA-DR/low cells that displayed TGF-β-mediated suppressive functions on T-cell activities.	(64)
Colorectal cancer-exosome	Clinical, *in vitro*, and *in vivo*	T cell	RORγt	Th17 differentiation	Exosomes isolated from colorectal cancer contained high levels of CRNDE-h, which it delivered into CD4+ T cells in co-culture, thus increasing the percentage of Th17 and upregulating RORγt expression and IL-17 promoter activity. CRNDE-h binds to the PPXY motif of RORγt and inhibits its ubiquitination and degradation by disrupting its interaction with the E3 ubiquitin ligase Itch.	(65)
Prostate cancer-exosome	*In vitro* and *in vivo*	Myeloid-derived suppressor cell (MDSC)	TLR2/NF-κB	Recruitment	Prostate cancer cell-derived exosomes upregulated CXCR4 expression in MDSCs through the activation of the TLR2/NF-κB, further facilitating the MDSCs’ migration into TME through CXCR4-CXCL12.	(66)
Breast cancer-exosome	Clinical, *in vitro*, and *in vivo*	MDSC	JAK/STAT	Expansion	Tumor-derived exosomes with miR-9 and miR-181a activated JAK/STAT by targeting SOCS3 and PIAS3 to promote MDSCs expansion.	(15)
T-cell acute lymphoblastic leukemia-exosome	Clinical and *in vivo*	T cell	Notch	Expansion/survival	Exosomes secreted by T-ALL harbor NOTCH-dependent miRNAs targeting oncogenic pathways, which act in an autocrine fashion to promote the expansion and survival of most proliferative cell subsets in human T-cell leukemias.	(67)
Lung adenocarcinoma-derived exosome	Clinical, *in vitro*, and *in vivo*	Macrophage	miR-3153/JNK	M2 polarization	Exosomal miR-3153 targets zinc finger protein 91 to inhibit the ubiquitination and degradation of misshapen-like kinase 1, which activates JNK and promotes M2 polarization.	(68)
Colon Cancer-Derived Exosome	*In vitro*	Macrophage	AKT, ERK, and STAT3/6	M2 polarization	Exosomal lncXIST promoted M2 polarization by modulating the miR-17-5p/PDGFRA axis.	(69)
Glioma-derived exosome	*In vitro* and *in vivo*	Macrophage	PI3K-AKT-mTOR	M2 polarization	miR-25-3p was upregulated in exosomes from hypoxic glioma cells and transferred into macrophages, downregulating PHLPP2 expression. The process activates PI3K-AKT-mTOR, promoting M2 polarization.	(70)
Breast cancer-derived exosome	*In vitro*	Macrophage	miR-138-5p/KDM6B	M2 polarization	miR-138-5p was transferred from breast cancer to TAMs via exosomes, downregulating the expression of KDM6B, suppressing M1 polarization, and promoting M2 polarization.	(71)
Osteosarcoma exosome	*In vitro*	Macrophage	TGFB2	M2 polarization	Osteosarcoma-exosomes facilitate M2 polarization in the tumor microenvironment by upregulating TGFβ2 signaling.	(72)
Ovarian cancer-exosome	*In vivo*	Macrophage	CCL13–CCR2/PI3K-Akt	M2 polarization	Exosomal SNHG17 upregulated the expression of CCL13 in M2 macrophages through PI3K-Akt.	(73)
Prostate cancer-exosome	*In vivo*	Macrophage	CXCL14-NF-κB	M2 polarization	Exosome-mediated CXCL14 caused the M2 polarization through NF-κB.	(74)
Gastric cancer-derived exosome	Clinical, *in vitro*, and *in vivo*	Macrophage	miR-541-5p-DUSP3/JAK2/STAT3	M2 polarization	Gastric cancer promoted M2 polarization by secreting exosomal miR-541-5p. This exosomal miRNA sustained the activation of JAK2/STAT3 in macrophages by targeting the negative regulator DUSP3.	(75)
Gastric cancer-derived exosome	Clinical and *in vitro*	Neutrophil	NF-Kb-HMGB1/TLR4	N2 polarization	Exosomes originating from gastric cancer have been shown to enhance autophagy and trigger a pro-tumorigenic response in neutrophils through activation of the HMGB1/TLR4/NF-κB	(76)
Melanoma-derived exosome	*In vitro* and *in vivo*	Macrophage	let-7a-Akt-mTOR	Metabolic reaction	Exosomal delivery of let-7a enhanced oxidative phosphorylation (OXPHOS) and promoted M2-polarization in infiltrating macrophages by suppressing the AKT-mTOR.	(77)
Lung cancer-derived microparticle	*In vivo*	Macrophage	mTORC1	Metabolic reprogramming	The uptake of tumor-derived microparticles by macrophages induced metabolic and phenotypic shift characterized by increased OXPHOS, elevated ATP production, and upregulated adhesion molecule expression through mTORC1-dependent reprogramming.	(78)
Non-small-cell lung cancer (NSCLC) derived exosome	*In vivo*	Macrophage	NF-kB	Glycolytic-dominant metabolic reprogramming	Tumor-exosomes induced an immunosuppressive phenotype in premetastatic niche macrophages through NF-kB-dependent, glycolysis-dominant metabolic reprogramming, which supports tumor metastasis.	(11)
Pancreatic cancer-exosome	Clinical and *in vitro*	Monocyte	STAT3	Immune suppression	Tumor-derived exosomes mediated immune suppression of monocytes via altered STAT3, increased expression of arginase, and levels of reactive oxygen specie (ROS).	(79)
Renal cancer-derived exosome	*In vivo*	MDSC	TLR2-HSP70	Immune tolerance	Renal cancer-derived exosomes induced immunosuppression mediated by MDSCs, and HSP70 was indispensable for this process.	(80)
Colorectal, bladder, and breast cancer-derived exosome	Clinical and *in vitro*	T cell	CD73/CD79	Suppression	Exosomes isolated from different types of cancer expressed significant ATP and 5′AMP phosphohydrolytic activity. Such activity was in part explained by the expression of CD39 and CD73 on exosomes.	(81)
Melanoma-extracellular vesicle (EV)	*In vivo*	MDSC	PD-L1/TLR4	Suppression	Melanoma-derived EVs, by incorporating inducible heat shock protein 86 (HSP86), activate TLR4 on myeloid cells, which in turn activates NF-κB to upregulate the expression of PD-L1.	(12)
Gastric cancer cell-derived EV	Clinical and *in vitro*	Neutrophil	HMGB/STAT3	T cell Suppression	Exosomes from the gastric cancer microenvironment increase PD-L1 expression on neutrophils and suppress T-cell immunity.	(46)
Prostate cancer-exosome	*In vitro* and clinical	DC	PGE2-CD73	Suppression	Prostate cancer-exosomes stimulated the expression of CD73, an ecto-5′-nucleotidase that converts AMP into adenosine, on CD39-positive dendritic cells, leading to ATP-mediated suppression of TNFα and IL-12 secretion.	(82)
Prostate cancer-exosome	*In vitro*	Macrophage	PD-L1-PI3K/AKT	Suppression	Prostate cancer exosomes altered the immunosuppressive microenvironment and affected macrophage polarization by enhancing the expression of PD-L1, inflammatory cytokines and activating PI3K/AKT.	(83)
Head and neck squamous cell carcinoma (HNSCC)-derived exosome	Clinical	T reg cell	Ca2+ influx	Treg suppressor	Exosomes mediated Ca2+ influx in T cells purified from the plasma of cancer patients, delivering robust and sustained signaling to Treg cells. The prolonged signaling in Treg cells resulted in a striking increase in the conversion of extracellular ATP to inosine by Treg cells, indicating that signaling may have functional implications on these recipient cells.	(84)
Ovarian cancer-microvesicle	Clinical and *in vitro*	T reg cell	STAT3	Treg suppressor	TDEV mediated Treg cells expansion and up-regulated their suppressive functions via mechanisms involved IL-10 and TGF-β1.	(85)
Lung carcinoma-derived microvesicle	*In vitro* and *in vivo*	NK cell	TGF-b1/NKG2D	Suppression	Hypoxic tumor-derived microvesicles, once internalized by NK cells, can deliver TGF-β1 that downregulates the surface expression of the activating receptor NKG2D, leading to impaired NK-cell activity.	(86)
ALL-derived exosome	*In vitro*	NK	TGF-β	Evade immune surveillance	Leukemic exosomes in ALL inhibit NK-cell proliferation and cytotoxicity, preventing the release of cytotoxic granules from these cells by TGF-β. This may enable a tumor to evade innate immune surveillance.	(87)
Ovarian cancer-derived exosome	*In vitro*	Monocytic precursor cells	TLR2/TLR4-NFkB/STAT3	Inflammatory cytokines	THP-1 cells internalized exosomes, which induced the production of IL-1β, TNF-α, and IL-6 through the activation of NF-κB and further initiated the activation of STAT3.	(13)
Breast cancer-derived exosome	*In vivo*	Macrophage	TLR2/MyD88-NF-kB	Pro-inflammatory cytokine	The palmitoylated proteins exposed on the exosome surface confer the activation of TLR2, which triggers NF-κB activation and inflammatory cytokine expression in macrophages.	(14)
Colorectal cancer- and multiple myeloma-derived small EVs	*In vitro*	Macrophage	IL-6/STAT3-TLR4	Pro-Inflammatory Cytokine	SEV remarkably upregulated PD-L1 and IL-6 expression and activated STAT3, as well as TLR4/NF-κB, which contributed to SEV-mediated PD-L1 expression.	(88)
Breast cancer-derived EV	Clinical and *in vitro*	Macrophage	cGAS/STING	Pro-inflammatory signature	Triple-negative breast cancer (TNBC) cells release EVs that promote the differentiation of monocytes into various macrophage subsets, thereby boosting the pro-inflammatory interferon response via cGAS/STING.	(89)
HNSCC-derived EVs	*In vitro* and *in vivo*	Macrophage	NLRP3	Inflammatory response	Exosomes isolated from HNSCC inhibited the induction of pro-IL-1β and pro-caspase-1 proteins, as well as the expression of the NLRP3, in priming for the activation of the inflammasome.	(90)
Liver cancer-exosome	*In vitro*	Macrophage	STAT3	Cytokine secretion	Exposure to liver cancer-exosomes linked with endoplasmic reticulum stress resulted in suppression of the JAK2/STAT3. Furthermore, treatment of RAW264.7 cells with the STAT3 inhibitor S3I-201 significantly decreased cytokine release.	(91)
Multiple myeloma- exosome	*In vitro*	NK cell	HSP70/TLR2/NF-kB	Cytokine production	Several myeloma-derived exosomes activated the IFN-γ production in NK via a mechanism depending on the activation of NF-kB in a TLR2/ HSP70-dependent manner.	(92)
HNSCC-microvesicle	Clinical and *in vitro*	CD8 T cell	TCR and IL-2R	Apoptosis	HNSCC-derived microvesicles inhibited signaling and proliferation of activated CD8^+^ T cells and induced apoptosis, including in tumor-reactive tetramer+CD8^+^ T cells.	(93)
Lung adenocarcinoma, hepatocellular carcinoma, and breast cancer- exosome	*In vitro*	Monocyte	MAPK/Receptor tyrosine kinase (RTK)	Apoptosis	This study identified a tumor-associated monocyte survival-promoting mechanism by which exosomes derived from cancer cells activated the MAPK in monocytes by transferring functional RTKs into monocytes, resulting in the inhibition of apoptosis-related caspases.	(94)
Pancreatic cancer-derived exosome	*In vitro*	T cell	p38 MAPK	Apoptosis	T cells internalizing pancreatic cancer-derived exosomes underwent apoptosis through ER stress mediated by the activation of p38 MAPK.	(95)
Ovarian cancer-exosome	Clinical	T cell	JAK-3	Apoptosis	Ovarian cancer exosomes inhibited the expression of key T-cell activation components, CD3-ζ and JAK3, and induced apoptosis.	(96)

### 4.1 Recruitment, differentiation, and expansion

During cancer progression, immune cells migrate from the bloodstream into the TME, where they trigger inflammation and contribute to the spread of the tumor (97). EVs secreted by pancreatic ductal adenocarcinoma (PDAC) promote the recruitment of bone marrow-derived cells, such as macrophages, to the liver by stimulating Kupffer cells to increase their secretion of TGF-β. EVs derived from myeloma cells that contain serglycin enhance macrophage migration more effectively than EVs lacking serglycin, following their uptake by the macrophages (98). Moreover, EVs derived from the breast cancer with high expression of signal-induced proliferation-associated 1 (SIPA1) enhance macrophage migration toward the tumor site (99). Recent findings show that exosome-like EVs promote dendritic cell (DC) migration by enhancing their response to chemokine (C–C motif) ligand 19 (CCL19) and CCL21 (100). This effect is mediated by membrane-bound C-X3-C motif chemokine ligand 1 (CX3CL1) on endothelial EVs, which activates G-protein-coupled receptor (GPCR) through the CX3CR1, inducing dynamic protrusions and directional movement toward CCL19 (100).

Genetic deletion of TLR2 in macrophages found to abolish NF-κB activation triggered by breast cancer secreated exosomes (14). Li et al. (66) demonstrated that tumor-derived exosomes enhance NF-κB activation and upregulate CXCR4 expression in MDSCs, promoting their expansion and migration (66). This effect was significantly reduced by preincubation with the TLR2 inhibitor C29, suggesting that exosomes act via TLR2 to activate NF-κB signaling and subsequently elevate CXCR4 levels. Another experiment demonstrated that tumor-derived exosomes may enhance CXCR4 expression, potentially through the increased phosphorylation of NF-κB, leading to the expansion and migration of MDSCs (66).

An increased frequency of circulating CD4^+^CD25^+^FOXP3 (forkhead box P3)^+^ Tregs is commonly observed in cancer patients (101). TDEVs have been shown to promote the transformation of human conventional CD4^+^CD25^–^ T cells into CD4^+^CD25^+^FOXP3^+^ Tregs in a TGF–β1–dependent manner, resulting in elevated levels of phosphorylated Suppressor of Mothers Against Decapentaplegic (SMAD) 2/3 and phosphorylated STAT3 and enhanced Treg proliferation (85, 93). Moreover, Tregs co-cultivated with TDEVs expressed higher levels of FasL, TGF-β, IL-10, cytotoxic T-lymphocyte antigen 4 (CTLA4), granzyme B, and perforin, and stronger suppressor functions (85). Additionally, Tregs that proliferated in response to TDEVs exhibited complete resistance to TDEV-mediated apoptosis (85, 102). Recent studies have also reported similar effects of TDEV on enhancing Treg activity (102).

Sun et al. (65) reported that exosomes from CRC carry the long non-coding RNA Colorectal Neoplasia Differentially Expressed (CRNDE)-h and transfer it to CD4^+^ T cells, which in turn promote T helper (Th) 17 differentiation. They found that CRNDE-h might inhibit the ubiquitination and degradation of retinoic acid-related orphan receptor gamma t (RORγt) by blocking its interaction with Itch, thus promoting Th17 differentiation in CRC. RORγt is a transcription factor for Th17 differentiation; it binds to the IL-17 promoter to induce secreted IL-17 and promotes the naive CD4^+^ T cells differentiation of into Th17 (103, 104). The results of Sun et al. (65) demonstrated that CRNDE-depleted CRC exosomes failed to enhance RORγt expression and IL-17 promoter activity, in contrast to normal CRC exosomes. Assays demonstrated that CRNDE binds to the PPXY motif of RORγt. These findings suggest that CRNDE-h, delivered by CRC-derived exosomes, enhances RORγt expression by suppressing its ubiquitination and degradation mediated by the E3 ubiquitin ligase Itch. Furthermore, it has been proposed that natural killer (NK) cell differentiation is contingent upon IL-15, which significantly contributes to NK cell proliferation and enhances NK cell survival (105, 106). Szczepanski et al. (107) found that exogenous IL-15 safeguards NK from the inhibitory influence of tumor-microvesicles via modulating SMAD signaling and that IL-15 can avert microvesicle-induced down-regulation of NK group 2D (NKG2D) expression. Recent findings indicate that IL-15 serves as a potent antagonist of TGF-β by obstructing the production of SMAD-DNA complexes, hence mitigating the down-regulatory effects on T cells (107).

Valenti et al. (64) discovered a pathway in which human tumor cells release membrane vesicles that influence monocyte differentiation into DCs, thereby promoting the generation of myeloid cells. Monocytes treated with tumor-derived microvesicles in the presence of granulocyte-macrophage colony-stimulating factor (GM-CSF) and IL-4 retained CD14 but showed reduced surface expression of HLA II, while costimulatory molecules CD80 and CD86 were not upregulated (64). The CD14^+^HLA-DR phenotype that resulted was linked to suppressive activity on various T-cell functions in response to T-lymphocyte receptor (TCR) triggering, such as proliferation, and expression of cytotoxic molecules, and Interferon gamma (IFN-γ). Valenti et al. (64) provide evidence that tumor cells in cancer patients may promote an immunosuppressive microenvironment by altering the differentiation pathway of circulating monocytes. Instead of developing into fully mature immune cells, these monocytes are redirected toward a CD14^+^ immature myeloid subset characterized by TGF–β–dependent suppressive functions, which impair T-cell proliferation and activity.

In HCC, exosomal HMGB1 has been shown to stimulate B cells with immunosuppressive properties that inhibit CD8^+^ T cell responses. This process also facilitates the expansion of Bregs by the TLR2/4 and mitogen-activated protein kinase (MAPK) signaling (51). Shi et al. (46) demonstrated that gastric cancer-derived EVs shuttle HMGB1, which activates STAT3 and upregulates PD-L1 on neutrophils that suppress T-cell proliferation, activation, and function. Notably, blocking the HMGB1 pathway only partially reduced PD-L1 expression, suggesting that TDEVs may also engage additional immunosuppressive mechanisms (46).

### 4.2 Immune cell polarization

Within the TME, TDEVs play a pivotal role as communicators (108). Through the transmission of several bioactive chemicals, TDEVs alter the immune cell polarization and activity in a way that promotes tumor growth (109). In this context, tumor-derived miR-103a in exosomes was demonstrated to directly modulate the macrophage polarization Phosphatase And Tensin Homolog (PTEN) by binding to its untranslated regions (UTRs), ultimately resulting in reduced expression (110). The downregulation of PTEN activates the PI3K/Akt and STAT3 signaling, resulting in increased accumulation of cancer-promoting molecules like IL-10, CCL2, and VEGF-A, while simultaneously reducing the antitumor immune response (110). Recent studies indicated that oral squamous cell carcinoma (OSCC) cells secrete a substantial quantity of circulating exosomes, abundant in miRNAs (111, 112). Studies have reported increased levels of miR-29 in developing OSCC tissues (112–114). When OSCC-derived exosomes were co-cultured with naïve macrophages, they induced M2 polarization, as indicated by elevated expression of CD206, Arg1, and IL-10. This effect was mediated by exosomal miR-29a-3p, which suppressed SOCS1 expression and promoted STAT1 phosphorylation, involving in the immunosuppressive TME (111). Cai et al. (112) found that tumor-exosomal miR-23a-3p induced M2 polarization by activating the SOCS1/STAT1 pathway, thereby facilitating OSCC cell proliferation and invasion. Similarly, coculture experiments using nonpolarized macrophages and miRNA-containing exosomes derived from normoxic or hypoxic epithelial ovarian cancer (EOC) cells elicited macrophage polarization into tumor-supportive M2 phenotypes, further supported by evidence of increased expression of M2 markers such as CD163 and CD206 (112). These miRNA-enriched exosomes were internalized by macrophages and regulated the SOCS/STAT pathway in these cells (112). Exosomes derived from EOC, enriched with miR-222-3p, were shown to downregulate SOCS3 expression and promote STAT3 phosphorylation in macrophages, enhancing the expression of M2 markers including CD206, Arg1, and IL-10 and decreasing pro-inflammatory cytokines TNF-α and IL-12, thus promoting the differentiation of macrophages into the M2 phenotype (115).

Lin et al. (116) found that exosomes derived bladder cancer can polarize M0 macrophages into an M2phenotype, evidenced by the secretion of particular cytokines and alterations in surface marker phenotypes. Their findings indicate that the exosomes produced from cancer cells initiate the polarization mechanism in macrophages. Lin et al. (116) further demonstrated that reduced expression of miR-21 in macrophages inhibits the polarization of M0 macrophages into the M2 phenotype. Conversely, overexpression of miR-21 in M0 macrophages was showed to enhance STAT3 expression, an effect reversed by miR-21 suppression. Additionally, the STAT3 upregulation induced by miR-21 inhibition was diminished upon treatment with SF1670, suggesting that this regulatory effect is dependent on PI3K/Akt pathway activation (116). Research indicated that augmenting the NF-κB in macrophages can increase the synthesis and secretion of IL-6 in cells (117). Recent evidence indicates that IL-6 and STAT3 are pivotal in the advancement of ovarian cancer, potentially through the polarization of TAMs (118).

Exosomal circFARSA (a circular RNA derived from the phenylalanyl-TRNA synthetase subunit alpha (FARSA) gene) generated from non-small-cell lung cancer (NSCLC) cells facilitates M2 polarization by enhancing PTEN ubiquitination and degradation, hence activating the PI3K/Akt pathway (119). The exosome derived circFARSA produced by NSCLC cells increased macrophage polarization towards the M2 phenotype, hence facilitating epithelial-mesenchymal transition (EMT) and migration. Chen et al. (119) discovered that exosome derived circFARSA, promotes PTEN ubiquitination and degradation in macrophages, consequently, facilitate macrophage polarization toward the M2 phenotype and activating the PI3K/Akt signaling.

Deng et al. (120) investigated the mechanism through which the exosome circATP8A1 facilitated M2 polarization. They revealed that STAT6 was activated following exosome treatment of macrophages, highlighting the significant involvement of STAT6 in gastric cancer (120). Prior research indicated that IL-4 facilitates M2 polarization primarily via STAT6 activation (121). M2 polarization entails tyrosine phosphorylation and STAT6 activation, which subsequently facilitates the transcriptional activation of M2 -specific genes, including Arg1 and mannose receptor 1 (Mrc1) (122). The findings of Deng et al. (120) validated that exosomal circATP8A1 produced from gastric cancer cells facilitates M2 polarization by influencing the STAT6. The ceRNA mechanism is a crucial pathway by which circRNAs exert their biological effects. The findings validated that the exosomes produced from gastric cancer cells, specifically circATP8A1, facilitate M2 macrophages polarization by competitively binding to miR-1-3p, hence activating the STAT6.

Tian et al. (74) demonstrated that exosomal CXCL14 facilitated M2 polarization via exosome-derived communication by NF-κB activation. As a member of the chemokine family, CXCL14 is essential for DC maturation, the induction of MHC I, immune cell infiltration, and cellular mobilization (123). Tian et al. (74) identified a strong correlation between the overexpression of CXCL14 and the expression of two M2 macrophage-associated markers, PD-L1 and IL-10, indicating CXCL14’s possible function in modulating M2 macrophage polarization. These findings indicated that CXCL14 facilitates macrophage polarization toward an M2-like phenotype. These findings demonstrated that NF-κB signaling was involved in the function of CXCL14 in M2 macrophage polarization in prostate cancer.

Research indicates that malignancies can elicit a pro-tumor phenotype in neutrophils (124). In a study by Zhang et al. (76), exosomes produced from gastric cancer cells stimulated autophagy in neutrophils by activating the NF-κB pathway via the HMGB1/TLR4 connection. Their findings elucidate a novel method for neutrophil regulation inside the TME and furnish additional data regarding the significant roles of exosomes in this context (76). G-CSF has been shown to activate autophagy in neutrophils, and the growth of neutrophils produced by G-CSF is compromised in the absence of autophagy (125). The findings indicate that exosomes derived from gastric cancer stimulated autophagy in neutrophils, resulting in the secretion of substances that enhanced the migratory capacity of gastric cancer. In summary, gastric cancer-exosomes promote autophagy and N2 neutrophil polarization via the HMGB1/TLR4/NF-кβ signaling.

### 4.3 Immunosuppression and immune evasion

TDEVs are essential for the ability of cancer cells to evade immune surveillance (126). These vesicles function as carriers of immunomodulatory molecules, thereby modifying the TME to promote immune evasion and suppress anti-tumor immunity (127). Previously, it was found that tumor-derived exosomes contain molecules from the TNF receptor (TNFR) superfamily, including FasL, which disrupt the function of T cells (96). Söderberg et al. (128) identified the presence of TNFR1 and TNFR2 in their study. Notably, TNFR2 has emerged as a key player in T cell activation and is thought to contribute to the tumor’s strategy to evade immune responses, a phenomenon often referred to as the tumor’s counterattack (129). Furthermore, the production of hydrogen peroxide (H_2_O_2_) in lymphocytes that internalized the exosomes highlights the functional importance of these vesicles (130, 131). Several lymphocyte receptors, including FasL, dopamine receptors, TLRs, and B-cell receptors, produce H_2_O_2_ as a signaling mediator (130, 131). Several studies have reported that reactive oxygen species (ROS) can lead to functional downregulation of TCR signaling by inactivating the CD3 zeta-chain (132). Melanoma-derived TNF displays dual functionality, acting as both a cytokine and growth factor by transferring to nearby melanoma cells via paracrine or juxtacrine routes, thereby activating NF-κB–mediated survival pathways, akin to IL-15 and IL-10 receptor signaling (133, 134). Exosomes play a key role in inducing oxidative stress, a condition recently shown to disrupt TCR signaling and reduce T cell numbers (135). The role of TNF in the TME of malignant melanoma may be partly explained by its involvement in promoting the secretion of ROS-generating exosomes, which contribute to immune evasion.

Chalmin et al. (53) discovered that MDSC expansion and activation are mediated by distinct molecular pathways. The ligand responsible for the activation and enhancement of the suppressive ability of TDEVs is Hsp72, which binds to TLR2 on MDSCs. Chalmin et al. (53) demonstrated that TDEVs possess immunosuppressive properties, which are activated by STAT3 phosphorylation in MDSCs. Their findings revealed that TDEVs trigger both STAT3 activation and the development of suppressive activity in MDSCs. Interestingly, the absence of PGE2 in these vesicles suggests that the Hsp72/TLR2 signaling axis may represent a previously unrecognized mechanism responsible for MDSC activation.

Jiang et al. (15) demonstrated that cancer exosome-miR-9 was strongly positively correlated with miR-181a and further upregulated miR-181a expression in MDSCs. Both miR-9 and miR-181a directly targeted SOCS3 and PIAS3, which are critical negative regulators of the JAK/STAT, leading to their suppressed expression. Notably, PIAS3 was upregulated when miR-181a levels were reduced and suppressed in MDSCs transfected with miR-181a mimics. The positive concordance observed between SOCS3 and PIAS3 in MDSCs hinted that miR-9 and miR-181a might act in a synergistic way. Jiang et al. (15) suggested that miR-9 and miR-181a independently regulated SOCS3 and PIAS3, they could coordinate with each other by regulating a synergetic ceRNA network to maintain the activation of the JAK/STAT.

Exosomes have an inhibitory effect on the maturation of DCs and the secretion of cytokines, as well as the differentiation of monocytes to DCs (82). DU145 exosomes drive DCs to express CD73 on their surface, creating a unique subset of DCs co-expressing CD39 and CD73. This phenotypic change allows the hydrolysis of adenosine triphosphate (ATP) into adenosine in the pericellular environment by these cells, thus contributing to immunosuppression (82). Adenosine has been demonstrated to have angiogenic, and metastasis-inducing effects under normal conditions. Additionally, it is a potent inhibitor of antitumor immune effector cells in the TME (136, 137). Salimu et al. (82) demonstrated that PGE2 is primarily found in the exosomal fraction of DU145 cell supernatants. They also found that the presence of ATP significantly reduced the ability of DCs treated with exosomes to secrete proinflammatory cytokines such as TNFα and IL-12 (82). These cytokines are essential for promoting anti-tumor immune responses, supporting DC maturation, and enhancing T cell activation (82). Inhibition of the PGE2 receptors EP2 and EP4 on DCs significantly reduced exosome-induced CD73 expression, suggesting that exosomal PGE2 engages in ligand–receptor interactions with DCs (138). PGE2 plays a critical role in upregulating C-C chemokine receptor type 7 (CCR7) and MMP-9 on DCs, thereby promoting their migration to lymph nodes (139, 140). PGE2 is a key driver of the expression of indoleamine 2,3-dioxygenase 1 (IDO1) on DCs, which attracts and induces Tregs. This would then lead to the suppression of CD8^+^ T cell responses—most likely creating an immunosuppressive environment (141). Salimu et al. (82) demonstrated that exosomal PGE2, through the induction of CD73 on DCs, played a role in the modulation of cytokine formation and T-cell activation. Notably, they assessed T-cell responses and showed that exosome-treated DCs suppressed T-cell activity in an adenosine-dependent manner, with the inhibitory effect intensifying in the presence of extracellular ATP or AMP.

TGF-β is a potent immunosuppressive factor that promotes tumor immune evasion by enhancing regulatory T cell activity, inhibiting T cell function, reducing IFN-γ production, and impairing antigen presentation (142, 143). TGF-β has been recently identified as a potent down-regulator of NKG2D-activating receptors on the surface of NK cells (144). Berchem et al. (86) demonstrated that hypoxic TDEVs significantly downregulate NKG2D expression on NK, suggesting a mechanism by which hypoxic TDEVs impair NK cytotoxicity, potentially through the delivery of TGF-β. Their findings support the notion that TDEVs reflect the hypoxic state of tumor cells by transporting immunosuppressive molecules that compromise NK function (86). Notably, NK from draining lymph nodes of mice treated with hypoxic TDEVs exhibited reduced levels of CD107a, IFN-γ, and Granzyme B compared to those treated with normoxic TDEVs (86).

Vulpis et al. (92) showed that the formation of IFN-γ on CD56 high NK is induced by MM-derived exosomes and is mediated by TLR2 engagement. NF-kB is involved in the activation of numerous cytokine genes, including IFN-γ, and is one of the primary pathways that TLRs activate. In this sense, exosome-induced IFN-γ production was determined to be contingent upon NF-kB signaling. A recent study proposed a model in which breast cancer-exosomes activate TLR2-mediated NF-κB signaling in human macrophages, leading to cytokine production, supporting and aligning with these findings (14). TLR2 recognizes damage-associated molecular patterns (DAMPs), including HSPs, which typically function intracellularly as molecular chaperones but act as potent danger signals when extracellular or membrane-bound, triggering immune activation (145, 146). EVs can associate with extracellular HSPs, as demonstrated by Vulpis et al. (92), who found elevated levels of HSP70 on the surface of multiple myeloma-exosomes. This HSP70 was suggested to play a role in stimulating IFN-γ production by NK in response to the exosomes.

Research has shown that infiltrating neutrophils express high levels of PD-L1, which suppresses T cell activation and proliferation (147). Shi et al. (46) showed a high PD-L1 expression on neutrophils, promoted by the gastric cancer microenvironment, thus suggesting that tumor signals may regulate neutrophils toward an immunosuppressive phenotype. They further show that gastric cancer-derived exosomes induce immunosuppressive neutrophils with elevated PD-1 expression via the activation of STAT3 (46). In conclusion, these results indicate that TDEVs are central mediators of tumor immune evasion, employing complex mechanisms to reduce immunity.

### 4.4 Inflammatory signatures

Cancer has been identified as an inflammatory disease (148, 149). Additionally, the immune system and inflammation have garnered a significant quantity of attention in relation to the development of cancer (149). In cancer cells, these exosomes have been found enriched with certain biomolecules such as NF-κB, STAT3, and pro-inflammatory cytokines (e.g., TNF-α, IL-1β, and IL-6); this means cancer cell-derived exosomes recruit immune cells to target sites (150). Bretz et al. (13) found that malignant ascites-derived ovarian cancer exosomes bind to TLR2 and TLR4 on THP-1 cells, thus eliciting the secretion of pro-inflammatory cytokines like IL-1β, TNF-α, IL-and 6, IL-8 by activation of NF-κB- and STAT3-dependent signaling. Other findings found that exosomes derived from breast and gastric cancer stimulate the production of pro-inflammatory cytokines (G-CSF, IL-1β, IL-6, IL-8, CCL2, and TNF-α) in M1 macrophages in an NF-κB-dependent manner (14). Chow et al. (14) demonstrated that cancer-derived exosomes interact with TLR2, leading to NF-κB activation in monocytes and macrophages, thereby highlighting the role of TLR2 in mediating the cross-talk between inflammation and cancer development. Moreover, they showed that exosomes from breast cancer can induce a pro-inflammatory response in macrophages, further supporting the link between tumor-derived exosomes and inflammation-driven tumor progression (14). These results were previously showed that exosomes activate NF-κB signaling and promote the secretion of inflammatory cytokines in monocytic cells (13). More recently, it was shown that membrane-associated Hsp72 from tumor exosomes activates STAT3 in MDSCs through a TLR2/Myeloid differentiation primary response 88 (MYD88)-dependent pathway mediated by the autocrine production of IL-6 (53). TNF-α secretion by myeloid cells is induced by the activation of TLR2:TLR6 complexes by cancer-secreted vesicles, thereby promoting the growth of metastatic tumors (151). Bretz et al. (13) showed that the treatment of THP-1 cells with tumor-derived exosomes triggers the activation of NF-κB and STAT3, which results in the formation of pro-inflammatory cytokines. They showed that tumor-derived exosomes promote the production of pro-inflammatory mediators, where NF-κB activation leads to IL-6 production, a prerequisite for subsequent STAT3 activation in an autocrine or paracrine way. Bretz et al. (13) showed this effect is mediated by TLR2/4, with cytokine induction reduced by their inhibition. MYD88 deficiency abolished this response, confirming TLR-dependent signaling. Finally, EVs can both activate and inhibit inflammasomes (152). Bottino et al. (90) found that HNSCC-derived EV-enriched vesicular secretome (VSF) inhibited NLRP3 inflammasome activation by reducing caspase-1 and IL-1β secretion in response to nigericin. This inhibition was linked to blocked NF-κB-mediated priming and may be due to TGF-β-related proteins in VSF. These findings suggest a tumor immune evasion mechanism via inflammasome suppression.

### 4.5 Metabolic reprogramming of immune cells

TDEVs reshape the immune environment by metabolically reprogramming immune cells to support immune evasion and tumor growth (1). Morrissey et al. (11) found that tumor exosomes activate NF-κB to drive an M1 macrophage and upregulate PD-L1 through both direct promoter binding and metabolic changes. They identified a new mechanism whereby NF-κB regulates PD-L1 expression, not only by direct binding to its promoter but also via metabolic reprogramming. NF-κB activation triggered two distinct pathways that enhanced glycolysis in response to TLR2/MyD88 signaling after tumor-derived exosome ligation. First, it upregulated Glucose transporter 1 (Glut1) expression via activation of Hypoxia-inducible factor 1α (HIF-1α), which increased glucose influx into macrophages. Second, activated Nitric oxide (NO) synthase-2 (NOS2) increased NO production, which inhibited complexes III and IV of the electron transport chain, driving pyruvate conversion to lactate. Morrissey et al. (11) also demonstrated that exogenous lactate activates NF-kB, resulting in the expression of PD-L1 on macrophages. The uptake of lactate intracellularly is required to promote PD-L1 expression, as evidenced by the reduction of lactate-induced PD-L1 expression on macrophages with the addition of the Monocarboxylate Transporter 1 (MCT-1) inhibitor AZ3965. In summary, Morrissey et al. (11) demonstrated that tumor-derived exosomes metabolically reprogram tissue-resident macrophages, promoting an immunosuppressive phenotype in the pre-metastatic niche.

Park et al. (77) documented an elevated level of macrophage chemotaxis toward exosomes generated by hypoxic tumors. Furthermore, hypoxic exosomes induced an increase in M2 polarization of myeloid cells and facilitated a modification in the immunometabolic profile of macrophages to improve OXPHOS activity (153, 154). These results are in accordance with observations that M2 macrophages promote tumor proliferation and increase angiogenesis by OXPHOS (155). Exosome-induced upregulation in the activity of OXPHOS in bone marrow-derived macrophages was associated with mTOR pathway inhibition and may thus hint at the possibility that exosomes could regulate the Akt-mTOR pathway by mechanisms of inhibition (77). Park et al. (77) demonstrated that let-7a miRNA is an epigenetic tumor suppressor and also a possible inhibitor of insulin-mediated mTOR signaling pathways. They showed that microenvironmental hypoxia downregulated let-7a levels in cancer cells and simultaneously enhanced exosome release (77). Moreover, the exosomal transfer of let-7a miRNA caused downregulation of insulin-mediated Akt-mTOR signaling, consequently promoting OXPHOS activity and M2 polarization of infiltrating macrophages (77).

Kersten et al. (78) showed that tumor-derived vesicles acutely activate mTORC1 in macrophages, which in turn promotes metabolic programs defined by increased OXPHOS and promotes anti-metastatic activity in the lung. The catalytic subunit mTOR, shared by both mTORC1 and mTORC2 complexes, is a key regulator of metabolism and found to promote mitochondrial biogenesis and oxidative activity (156, 157). An elaborate analysis of the mTORC1 pathway showed an upregulation of phospholipases D1 and D3 (*Pld1* and *Pld3*) and genes involved in protein synthesis, such as *Eukaryotic translation initiation factor 4E-binding protein 1* (*Eif4ebp1)*, *Protein Phosphatase 2 Phosphatase Activator* (*Ptpa), and Ribosomal Protein S23* (*Rps23)*. In contrast, downregulation was noted for Prkab2, a gene encoding a positive regulator of the AMPK, an upstream negative regulator of mTOR, indicating repression of the AMPK in macrophages (158). Kersten et al. (78) showed that the uptake of tumor-derived microparticles activates mTORC1 signaling in macrophages, as demonstrated by the greatly increased levels of phosphorylated (p)4EBP1 and pS6, resulting in metabolic and phenotypic changes in non-resident macrophages in the early metastatic lung, which strongly depended on mTORC1 signaling.

Clayton et al. (81) found that cancer-derived exosomes convert ATP and AMP into adenosine, increasing adenosine levels in the TME and suppressing T-cell activity, highlighting a mechanism of immune evasion. They point out that the expression of catalytically active membrane-associated enzymes like CD73 is an intrinsic feature of exosomes since their release into the microenvironment enhances the surface area for enzyme activity severalfold. Clayton et al. (81) focused on the immunosuppressive consequences of exosomal CD39/CD73 in generating adenosine within the TME. This adenosine production initiates downstream signaling through the A2A and A2B receptors, which activates adenylyl cyclase and yields cAMP. Exosomes can sequentially hydrolyze ATP and AMP to produce adenosine, which acts on cAMP signaling in A2A receptor-positive cells to further support immunosuppression in the malignant microenvironment. Clayton et al. (81) also showed the impact of adenosine generation on T cell function and demonstrated that the production of inflammatory cytokines and the proliferative responses of T cells could be effectively suppressed by AMP as a substrate. This inhibition was eliminated by chemically inhibiting the hydrolytic function of CD73. In conclusion, these results suggest that exosome secretion by cancer is a mechanism that contributes to the increase in adenosine levels in the TME.

### 4.6 Immune cell apoptosis

TDEVs deliver molecular cargo that induces apoptosis in immune cells (159). In this regard, surface CD95 is expressed by CD8^+^ T lymphocytes in cancer patients, and a significant number of them express PD-1 (160, 161). Consequently, CD8^+^ T lymphocytes susceptible to apoptosis when TDEV carries the membrane form of FasL and PD-L1 (160, 161). The frequency of apoptosis-sensitive activated CD8^+^ T cells in cancer patients was correlated with the expression of these apoptosis-inducing molecules in TDEVs (162, 163). It is crucial to note that the disease stage and prognosis were significantly correlated with the spontaneous apoptosis of circulating CD8^+^ T cells (160, 162). TDEVs triggered apoptosis in activated CD8^+^ T cells, as evidenced by early membrane alterations such as annexin V binding, caspase-3 activation, cytochrome c (cyt c) release from mitochondria, disruption of mitochondrial membrane potential, and DNA fragmentation (164). Inactivated CD8^+^ T cells, the PI3K/Akt pathway is a critical target of TDEVs (164, 165). PTEN, a phosphatase known for its role in regulating the PI3K/Akt signaling pathway, was recently identified as part of the cargo carried by TDEVs, where it retains its phosphatase activity and functions within recipient cells (166). Incubating activated CD8^+^ T cells with TDEVs led to a significant, time-dependent decrease in Akt phosphorylation, accompanied by reduced expression of the anti-apoptotic proteins B-cell lymphoma (BCL)-2, BCL-xL, and MCL-1 (166). Simultaneously, TDEV increased the expression of the proapoptotic protein Bax (164, 167). Subsequently, TDEV induces apoptosis in activated CD8^+^ T cells by activating intrinsic and extrinsic apoptosis pathways (164).

Of note, the immune response to tumor cells can be inhibited by exosomes released by cancer cells, particularly via apoptosis in T lymphocytes (168, 169). Shen et al. (95) found that exosomes from pancreatic cancer could significantly induce apoptosis of peripheral T lymphocytes following their internalization. They used Gene Set Enrichment Analysis (GSEA) to describe essential signaling pathways in this process and observed changes in the gene expression profiling of exosome-treated T cells (95). Notably, genes related to apoptosis and ER stress, such as p38 MAPK, JUN, eukaryotic translation initiation factor 2A (eIF2A), Protein Kinase RNA-Like ER Kinase (PERK), C/EBP homologous protein (CHOP), and Activating transcription factor 4 (ATF4), were altered, suggesting their roles in the induction of apoptosis (95). Phosphorylation of p38 MAPK was found to be a key driver of T lymphocyte apoptosis via ER stress. RNA sequencing of exosome-treated T cells identified 177 differentially expressed mRNAs, with 151 upregulated and 26 downregulated (95). Of these, apoptosis-related and unfolded protein response (ER stress) gene sets were strikingly enriched, suggesting that the ER stress signals are transduced to the nucleus by PERK-eIF2α-ATF4-CHOP and ultimately promote apoptosis (170). Shen et al. (95) further showed that pancreatic cancer-derived exosomes upregulated the expression of ATF4, PERK, and CHOP, accompanied by increased phosphorylation of eIF2α, indicative of ER stress signaling activation. The pro-apoptotic effects of ER stress were demonstrated to be finely modulated by the MAPK signaling. More precisely, p38 MAPK was shown to interact with CHOP, and the p38 MAPK inhibitor SB203580 efficiently disrupted this interaction. Shen et al. (95) found the involvement of p38 MAPK in ER stress-induced apoptosis and showed that its inhibition by SB203580 (a potent inhibitor of p38 MAPK activity) reduced the apoptotic effects. Pancreatic cancer derived exosomes, through upregulation of JUN, activated p38 MAPK, which mediated ER stress and apoptosis in T lymphocytes (95). Evidence also supported that p38 MAPK further induce JUN expression in T lymphocytes to reinforce the apoptotic signaling cascade (171, 172). SB203580 was able to inhibit this action, intriguingly, while exosome treatment resulted in an upregulation of DUSP1, a phosphatase that negatively modulates the MAPK; SB203580 reduced its levels (95). The alteration in p38 phosphorylation was in accordance with the alteration in DUSP1 expression. The results of Shen et al. (95) suggest that DUSP1 may serve as a critical mechanism to mitigate the excessive activity of p38 MAPK. In conclusion, the findings demonstrate that pancreatic cancer-derived exosomes are taken up by T lymphocytes, alter their gene expression and cytotoxic function, and induce ER stress-mediated apoptosis through activation of the p38 MAPK.

Abusamra et al. (173) demonstrated that the Fas–FasL pathway was essential for the apoptosis of T-cells mediated by cancer exosomes. Cancers have been observed to exhibit an elevated expression of FasL (174). The Fas–FasL pathway has also been extensively used to document the apoptosis of tumor-reactive T cells (175). Abusamra et al. (173) indicated that “membrane-bound” FasL, which is transported by exosomes, is present in the peripheral circulation. This tumor-derived circulating FasL has the potential to efficiently induce apoptosis by targeting circulating T cells at a distance (175). These results demonstrated that exosomes expressing FasL suppressed T-cell responses by inducing apoptosis. Finally, Huber et al. (169) demonstrated that CRC triggered apoptosis in T cells via the release of FasL and TNF-related apoptosis-inducing ligand (TRAIL)-bearing microvesicles. The expression of FasL, a key molecule in CRC progression, emerges in the early stages of colon carcinogenesis, correlates with tumor spread, and is associated with increased apoptosis of lymphocytes within the TME (169). The secretory pathway of proapoptotic microvesicles, common to various tumor histotypes including melanoma, constitutes an effective mechanism for CRC to transmit death signals to antitumor T lymphocytes without requiring direct cell-to-cell interaction. Collectively, the evidence underscores the pivotal role of TDEVs, including exosomes and microvesicles, in mediating immune evasion through the induction of T cell apoptosis. The findings highlight the multifaceted strategies employed by tumors to suppress antitumor immunity and emphasize the therapeutic potential of targeting TDEV-mediated immune suppression to restore effective T cell responses in cancer.

## 5 The function of TDEVs in immune signaling modulation caused tumor development, angiogenesis, and metastasis

One of the important roles of TDEVs is the regulation of immune signaling, which plays an essential role in tumor development, angiogenesis, and metastasis (**Table 3**). In this sense, TAMs, which are predominantly of the M2 phenotype, are significant contributors to pro-angiogenic activity in malignancies and, as such, play vital roles within the TME (191). TAMs involve in tumor angiogenesis by secreting several pro-angiogenic cytokines, including VEGFA, platelet-derived growth factor β (PDGF-β), angiogenin, placental growth factor (PlGF), TGF-β, and MMPs (192–195). In a coordinated manner, these factors promote the formation and remodeling of tumor-associated vasculature to feed tumor growth and progression (184, 196). Some works confirmed that the PI3K/AKT regulates numerous pro-angiogenic cytokines, including VEGFA and MMP9, which are involved in tumor angiogenesis (197). PTEN is a phosphatase that has the ability to dephosphorylate PIP3 into PIP2 and thereby directly inhibit the oncogenic PI3K/AKT (198). Esophageal squamous cell carcinoma (ESCC) cells secrete exosomes that induce M2 polarization through the PTEN/PI3K/AKT, as revealed by Shou et al. (184). Research has proven the role of TAMs in promoting tumor angiogenesis by showing that TAMs can enhance the migration, proliferation, and invasion of tumor endothelial cells (TECs) through the secretion of VEGFA and MMP9, contributing to the development and remodeling of tumor-associated vasculature to support angiogenesis and tumor progression. Shou et al. (184) identified miR-301a-3p as being significantly upregulated and demonstrated its positive correlation with Tumor, Node, Metastasis (TNM) stage, tumor invasion, and lymph node metastasis; therefore, it may regulate the pro-angiogenic switch of macrophages via PTEN/PI3K/Akt signaling. Their findings showed that exosomal miR-301a-3p derived from ESCC could induce M2 polarization via this pathway, thereby indirectly promoting angiogenesis and tumor progression.

**Table 3:** Tumor-derived vesicles and immune signaling modulation caused tumor development and metastasis.

Tumor-derived vesicle	Study	Immune cell	Signaling	Outcome	Conclusion	Ref.
Lung cancer microparticle	*In vitro* and *in vivo*	Macrophage	TLR3/NLRP3	Tumor development	Macrophages activated by lung cancer-derived microparticles secrete the proinflammatory cytokine IL-1β, which enhances lung cancer progression.	(176)
Breast cancer-exosome	*In vitro*	T cell	PD-1	Tumor growth	PD-L1, other than its passive protective role, is also actively involved in the defense mechanisms contributing to exosomes that dampen anti-tumor immunity within TME.	(177)
Gastric cancer-exosome	*In vitro*	Macrophage	NF-κB	Cancer progression	Gastric cancer-derived exosomes triggered NF-κB signaling in macrophages, thereby facilitating tumor advancement.	(178)
Glioma-derived exosome	Clinical and *in vitro*	Macrophage	JAK2/PI3K/AKT/mTOR, JAK2/STAT3, and MAPK	Cancer progression	Glioma-released exosomal miR-3591-3p promotes macrophage to the M2 phenotype by targeting CBLB to activate the JAK2/PI3K/Akt/mTOR and STAT3, thereby promoting glioma progression.	(179)
Lung cancer-exosome	*In vitro* and *in vivo*	Macrophage	NLRP3	Cancer progression	Tumor-derived exosomal TRIM59 reprograms macrophages toward a tumor-promoting phenotype by inducing the proteasomal degradation of ABHD5, thereby activating the NLRP3 and enhancing lung cancer progression through increased IL-1β secretion.	(180)
Gastric cancer-exosome	Clinical and *in vitro*	Macrophage	miR-1-3p/STAT6	Cancer progression	Exosome-derived circATP8A1 from gastric cancer induces M2 polarization via the circATP8A1/miR-1-3p/STAT6 pathway, thereby facilitating tumor progression.	(120)
Lung cancer-exosome	*In vitro*	Macrophage	miR-124-3p/EZH2	Cancer progression	Lung cancer-secreted exosomal circPVT1 contributes to macrophage polarization modulation by downregulating miR-124-3p, thus facilitating the enhancement in lung cancer cells of proliferation, invasion, and migration by elevating EZH2 expression.	(181)
Bladder cancer−exosome	*In vitro*	Macrophage	PI3K/AKT	Cancer progression	Bladder cancer -derived exosomal miR-21 inhibits the activation of phosphatase and tensin homolog (PTEN) in macrophages, disturbing PI3K/AKT, which increases STAT3 expression. This induces M2 macrophage polarization, thus supporting cancer progression.	(116)
Osteosarcoma-exosome	Clinical, *in vitro*, and *in vivo*	Macrophage	ELFN1-AS1	Cancer progression	Osteosarcoma-exosomal ELFN1-AS1 promotes M2 polarization by sponging miR-138-5p and miR-1291; the derived M2 macrophages further significantly promoted osteosarcoma progression.	(182)
Breast cancer-exosome	Clinical, *in vitro*, and *in vivo*	Macrophage	PTEN/Akt	Cancer progression	Chemotherapy-resistant breast cancer uses exosomal miR-222 to reprogram macrophages in TME and, by targeting PTEN, initiates the Akt signaling cascade that promotes M2 macrophage polarization.	(183)
Esophageal squamous cell carcinoma-exosome	Clinical and *in vitro*	Macrophages	PTEN/PI3K/AKT	Angiogenesis	miR-301a-3p carried by exosomes drives M2 macrophage polarization by suppressing PTEN and activating the PI3K/AKT pathway, which in turn enhances angiogenesis via increased secretion of VEGFA and MMP9.	(184)
Small cell lung cancer (SCLC)-exosome	Clinical and *in vivo*	Macrophage	NLRP6/NF-κB	Metastasis	SCLC exosomes can promote M2 polarization through the NLRP6/NF-κB, which might contribute to metastasis in SCLC.	(185)
Ovarian cancer-exosome	*In vitro* and *in vivo*	Macrophage	AKT/mTOR	Metastasis	The expression of miR-205 is associated with TAMs infiltration in ovarian cancer. Moreover, ovarian cancer exosomal miR-205 induces M2 polarization by modulating the PTEN/PI3K/AKT/mTOR, thereby promoting the progression and metastasis of cancer cells.	(186)
Lung adenocarcinoma-exosome	*In vivo*	Macrophage	miR-19b-3p/Hippo	Metastasis	Exosomal miR-19b-3p from LUAD induces M2 in THP-1 cells via targeting of PTPRD/STAT3. In turn, STAT3-activated LINC00273 is shuttled by M2 macrophage-derived exosomes to LUAD cells where it activates YAP to facilitate RBMX-mediated packaging of miR-19b-3p into LUAD-derived exosomes.	(187)
Pancreatic cancer-exosome	Clinical, *in vitro*, and *in vivo*	Macrophage	PTEN/PI3Kg	Metastasis	Hypoxic exosomal miR-301a-3p promotes M2 polarization by activating the PTEN/PI3K to enhance their metastatic potential.	(188)
NSCL-exosome	Clinical and *in vitro*	Macrophage	PTEN/PI3K/AKT	Metastasis	Exosomal circFARSA promotes M2 polarization by facilitating ubiquitination and degradation of PTEN, which activates PI3K/AKT.	(119)
Glioma-derived exosome	Clinical and *in vitro*	Myeloid-derived suppressor cell (MDSC)	TGF-β1/miR-486-3p	Metastasis	Glioma-derived exosomes could shuttle AGAP2-AS1 into MDSCs, which enhanced the immunosuppression status of the host and promoted tumor progression.	(189)
B16 tumor-exosome	*In vitro* and *in vivo*	MDSC	MyD88	Metastasis	MyD88 was required for induction of IL-6 and TNF-α, activation of MDSCs, and tumor metastasis mediated by tumor-derived exosomes.	(190)

Wu et al. (178) found the function of exosomes in the activation of macrophages with regard to the NF-κB pathway. Their findings revealed that exosomes derived from gastric cancer could trigger NF-κB activation in human primary macrophages. This activation was significantly suppressed when macrophages were pretreated with an NF-κB inhibitor. To survey the function of exosome-activated macrophages on gastric cancer proliferation, migration, and invasion, Wu et al. (178) incubated MGC-803 cells with supernatant from cancer exosome-treated primary macrophages and the findings showed that the supernatant enhanced MGC-803 cell migration, colony formation, and invasion, effects that were reversed by NF-κB inhibitor pretreatment. Collectively, the study highlights that gastric cancer-derived exosomes activate macrophages through the NF-κB, thereby facilitating tumor progression.

Zhou et al. (199) revealed that mucosa-associated lymphoid tissue lymphoma translocation protein 1 (MALT1) was expressed in tumor but vastly expressed in TAMs in these patients with breast cancer with highly expressed cirRNA cSERPINE2. They demonstrated that tumor exosomal cSERPINE2 from breast cancer could upregulate MALT1 in TAMs, which then activated the NF-κB and increased the secretion of IL-6 by TAMs. Blocking IL-6 could reverse the tumor-promoting effect caused by cSERPINE2 overexpression. In addition, the TAM-secreted IL-6 stimulated the JAK2-STAT3 pathway in tumor cells and then upregulated the expression of CCL2 and EIF4A3. The IL-6 secretion upregulated by tumor exosomal cSERPINE2-educated TAMs enhanced CCL2 expression, and thereby, TAMS infiltration into TME was enhanced.

Menghisteab et al. (200) identified a novel mechanism through which CRC-derived EVs stimulate macrophages to generate MMPs and promote invasion, contributing to the formation of a pre-metastatic niche. While further research is needed to confirm the exact mechanisms, their findings clearly indicate that CD147 on CRC-EVs plays a key role in activating MMP12 in macrophages, thereby enhancing tumor cell invasion. The results of Menghisteab et al. (200) indicated that CD147^+^ EVs are involved in the activation of MAPK in macrophages, which results in the expression of MMP and the enhancement of tumor cell invasion. They emphasized that CRC-EVs are major contributors to promoting an invasion-prone phenotype in macrophages, possibly via CD147. This exosome population can significantly contribute to the activities of macrophages, including extracellular matrix (ECM) degradation, VEGF release, recruitment of more cells in the polymorphonuclear leukocytes (PMNs), and, finally, metastasis enhancement through ECM degradation and modulation of the PMN. These findings suggest that CRC-EVs could become essential mediators in the metastatic process by modulating macrophage behavior.

**Figure 3:**
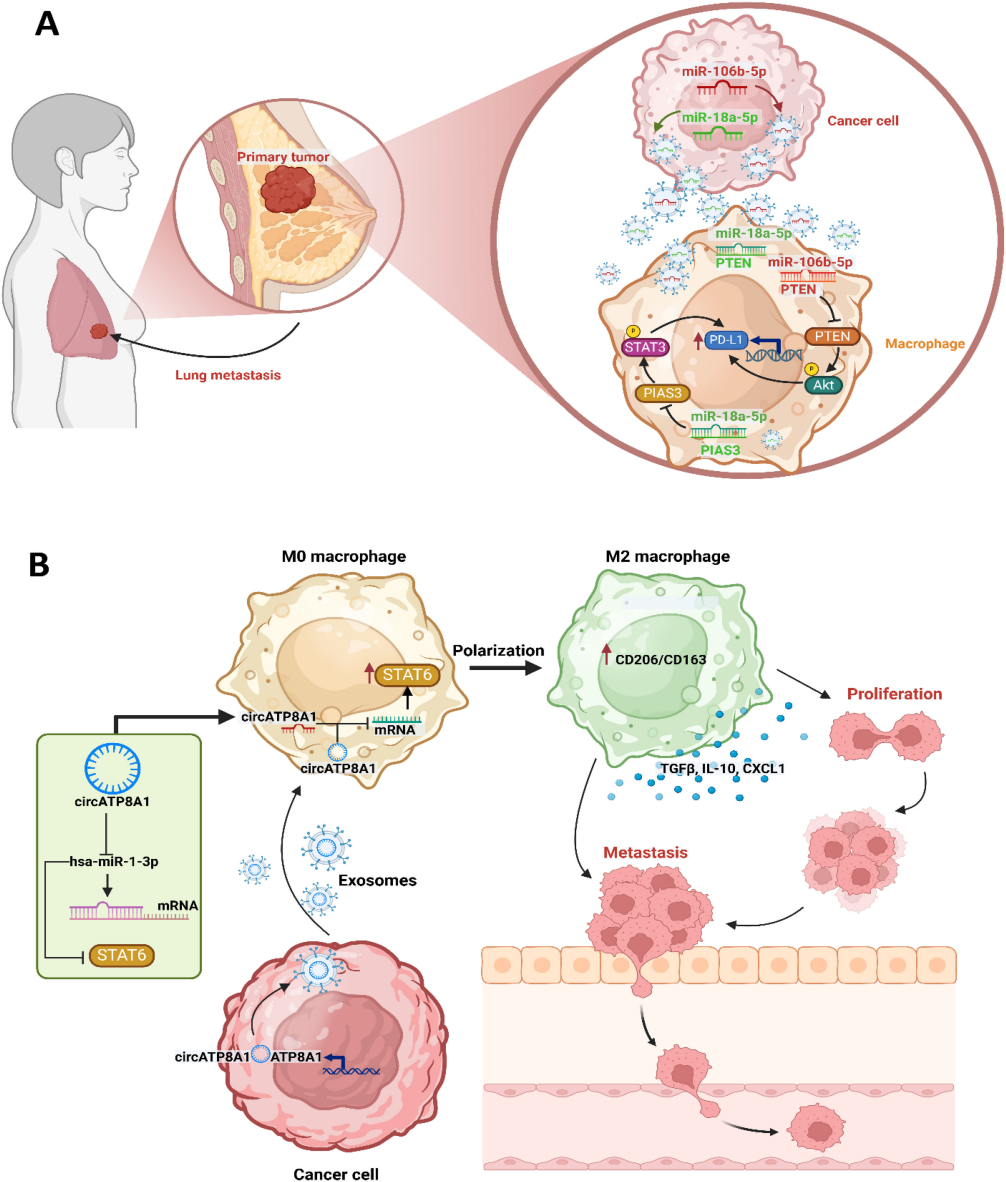
Immune signaling modulation by tumor-derived vesicles caused tumor development and metastasis. **(A)** In breast cancer, tumor-derived small EVs (sEVs) induce an immunosuppressive niche through the regulation of TAMs and increased expression of PD-L1. Especially, PD-L1 expression is higher in TAMs compared with cancer cells in breast, and a specific PD-L1^+^CD163^+^ TAM subpopulation is strongly associated with metastatic potential. Further, breast cancer-derived sEVs contribute to the establishment of this immunosuppressive environment by promoting PD-L1 expression and supporting M2 macrophage polarization, which allows immune evasion and leads to enhanced metastatic progression. Among the key molecular drivers of this process are sEV-associated miR-106b-5p and miR-18a-5p, whose expression is associated with poor prognosis and high metastatic potential in patients with breast cancer. In a synergistic manner, these miRNAs ensure the upregulation of PD-L1 in M2 TAMs by targeting the PTEN/AKT and PIAS3/STAT3. Through this orchestrated immune modulation, breast cancer cells acquire an invasive advantage by evading immune surveillance but favoring metastatic dissemination. **(B)** In gastric cancer, TDEV-mediated immune modulation is generally dominated by circATP8A1, a circular RNA that is highly enriched in exosomes derived from gastric cancer. Clinical studies have shown that high circATP8A1 levels correlate with advanced TNM staging and poor prognosis. Mechanistically, circATP8A1 acts through the induction of M2 macrophage polarization, associated with immunosuppression and tumor promotion. Unlike many other tumor-associated immune mechanisms that depend on STAT3, circATP8A1 specifically activates the STAT6 by competitively binding miR-1-3p. This sequestration of miR-1-3p blocks its inhibitory effects on STAT6, thus promoting M2 polarization. Functionally, exosome-treated M2 macrophages increase gastric cancer cell migration, which reinforces a pro-tumorigenic loop. Knockdown of circATP8A1 disturbs this axis and inhibits gastric cancer progression.

Rao et al. (185) demonstrated that exosomes secreted from small cell lung cancer (SCLC) cells can polarize macrophages toward the M0 to M2 phenotype. Such phenotype switching induced by SCLC-derived exosomes can partly explain one way that exosomes promote distant metastasis in SCLC because switching to the M2 phenotype in other tissues can initiate metastasis. Metabolic stimuli and inflammatory signals, including Peroxisome proliferator-activated receptor gamma (PPAR-γ) and TNF-α, have been shown to modulate NLRP6 expression at transcriptional and post-translational levels (201). Through the inhibition of IL-6 signaling, NLRP6 safeguards against the development of colon tumors associated with inflammation (202). Rao et al. (185) demonstrated that NLRP6, which normally inhibits tumorigenicity in gastric cancer and CRC, acts as a promoter of metastasis in SCLC and also its expression was significantly upregulated. In addition, their study of SCLC-derived exosomes showed that the inhibition of exosome release markedly inhibited NLRP6 expression and the M0-M2 switching response. These results underscore the association of SCLC-derived exosomes with the M0 phenotype switch and that activation of the NLRP6/NF-κB pathway facilitated SCLC metastasis. Taken together, TDEVs play a central role in establishing an immunosuppressive TME, hence allowing tumors to survive and develop (**Figure 3**). Also, TDEVs actively support angiogenesis and are likely to contribute to the preparation of pre-metastatic niches, thus positioning them as real drivers of tumor progression and metastasis.

## 6 Tumor-derived vesicles on immune signaling as a target for immunotherapy

TDEVs are under intense scrutiny in immunotherapeutic research due to several key attributes (203). First, their possible role in antigen presentation supports the development of anti-tumor vaccination strategies (204). Second, TDEVs’ inherent immunosuppressive properties shed light on tumor-immune interactions (59, 205). Third, based on their ability to be internalized by living cells, TDEVs become excellent biological carriers for delivering engineered therapeutic agents (206). Therefore, targeting key transcriptional networks regulating M2-polarized macrophages is a novel approach to reprogram the genetic profile of TAMs directly (207). STAT6 was selected for these experiments because of its pivotal role as a regulator of the macrophage M2 transcriptional program inside the TME across many malignancies (208). Besides its function in TAMs, STAT6 is crucial for CD4 Th2 responses and facilitates immunoglobulin (Ig) class switching to IgE and IgG1 in B cells, along with antigen presentation (209, 210).

Kamerkar et al. (207) revealed that exosomes facilitate the selective targeting of immune-suppressive TAMs and myeloid cells while minimally affecting lymphocytes and non-immune cells. In their study, augmented STAT6 suppression using an exosome therapeutic candidate delivering an antisense oligonucleotide (ASO) targeting STAT6 (exoASO-STAT6) led to significant macrophage reprogramming. ExoASOSTAT6 reprogramed macrophages in the presence of a combination of immunosuppressive cytokines such as TGF-β, IL-4, and IL-10 that often present in the TME (207). The administration of exoASO-STAT6 led to a significant reduction in the M2 macrophage phenotype and simultaneously, a strong activation of the M1 gene signature and proinflammatory cytokines was noted following exoASO-STAT6 therapy (207). The disparity between exoASO-STAT6 and free ASO is pronounced, evidenced by the significant tumor growth inhibition elicited by exoASO-STAT6, in contrast to the inactivity of free ASO at an equivalent dosage. Kamerkar et al. (207) presented the inaugural evidence of utilizing exosomes for the targeted delivery of ASOs, enhancing STAT6 knockdown in TAMs, and reprogramming macrophages amid immune-suppressive cytokines. Furthermore, the benefits of exosome-mediated delivery of ASO are evident *in vivo*, demonstrating that the effective antitumoral dosage of exoASO-STAT6 is 50–100 times lower than that reported in preclinical research involving untargeted ASO treatments (211). In conclusion, exosome-mediated delivery significantly enhances the potency of ASOs to inhibit STAT6 expression in TAMs and effectively reprograms them to an M1 phenotype. Consequently, exoASO-STAT6 therapy induces significant modification of the TME to a proinflammatory, antitumoral state and facilitates the formation of the CD8^+^ T cell-mediated response. The potency and specificity of exoASO-STAT6 yield significant single-agent antitumor efficacy, setting this innovative therapeutic strategy apart from conventional macrophage-targeting treatments and underscoring its clinical promise.

In addition to therapeutic delivery, TDEVs themselves modulate immune responses. During monocyte-to-macrophage differentiation, the downregulation of specific miRNAs leads to increased IKKα and NF-κB activation (212). Jang et al. (213) demonstrated that epigallocatechin gallate (EGCG) influences the tumor exosomes and enhances miR-16, which leads to the downregulation of IKKα and the inhibition of M2 polarization in TAM following EGCG treatment. Given that miR-16 may act as a tumor suppressor, it is plausible that elevated exosomal miR-16 could have influenced the survival and proliferation of tumor cells (213). NF-κB activation is recognized as a crucial factor for the differentiation of macrophages into M2 macrophages and has a role in tumor growth (214). Consequently, NF-κB in TAMs is regarded as a unique therapeutic target for cancer management. Jang et al. (213) noted the down-regulation of the NF-κB, specifically regarding IKKα and I-κB expression in TAM, induced by EGCG-treated exosomes (213). They identified this as the mechanism responsible for the EGCG-mediated inhibition of macrophage infiltration and differentiation into tumor-promoting M2 macrophages despite the absence of direct evidence.

It has been revealed that T-cells triggered by OKT3 (anti-CD3 antibody) incurred apoptosis, as seen by DNA fragmentation when treated with isolated fractions of TDEVs or CH-11 (anti-CD9) antibody, which cross-links Fas (213). The pretreatment of T cells with pan-caspase inhibitors [Z-VAD-FMK (benzyloxycarbonyl-Val-Ala-DL-Asp-fluoromethylketone)] slightly, however considerably, inhibited this effect (162). Only TDEVs, such as DCs or fibroblasts, triggered apoptosis in activated CD8^+^ T-cells (215). The capacity of TDEVs to provoke CD8^+^ T-cell apoptosis was attributed to the presence of membrane-bound FasL and MHC I in TDEVs (162, 163, 216). While the function of FasL in TDEV causing apoptosis in activated Fas^+^ CD8^+^ T-cells is unequivocal, the participation of MHC I remains conjectural. The direct interaction of MHC I with the CD8 receptor may activate the intrinsic Fas/FasL pathway, resulting in T-cell death (217, 218). The *ex vivo* investigations involving human T-cells and pure TDEVs unequivocally associate TDEVs with the decline of CD8^+^ T-cells noted in cancer patients, particularly pronounced following immunotherapy, during which CD8^+^ T-cells are stimulated and susceptible to apoptosis (217, 218). Kitai et al. (219) showed that topotecan (TPT) generates immunostimulatory DNA by TPT-mediated cell death, where the TPT-topoisomerase I-DNA complex activates the Stimulator of IFN genes (STING) pathway, likely through engagement with the DNA sensor cGAS. Kitai et al. (219) showed that TPT-treated cells activate DCs to produce inflammatory cytokines, chemokines, and type I IFNs in a STING-dependent manner (219). Moreover, TPT treatment elicited CD8^+^ T cell infiltration and activation at the tumor sites. Conditioned medium (CM) stimulation increased type I IFN production, STAT1 phosphorylation, and upregulation of cGAS, STING, and IFN regulatory factor 7 (IRF7). Type I IFN further stimulated DCs to upregulate the expression of genes, including 4-1BBL, CD30L, CD86, IL-6, IL-27, CXCL9, CXCL10, and CXCL11, suggesting that the cGAS-STING axis is essential in the immune responses elicited by TPT (219). TPT enhanced migration and expression of costimulatory molecules in unstimulated DCs but inhibited DC maturation induced by proinflammatory cytokines through suppression of NF-κB activity (219). Recently, it was demonstrated that TPT inhibits endotoxin-induced septic shock by suppressing pathogen-associated molecular pattern (PAMP)-induced inflammatory cytokine production by facilitating topoisomerase I-mediated nucleosome remodeling (220). In summary, Kitai et al. (219) showed that treatment with a high concentration of TPT only marginally increased IL-6 production by DCs. This may suggest that excessive TPT could suppress the innate immune activation either through the induction of excessive cell death and, as a result, a decrease in the availability of the immunostimulatory DNA or through the induction of topoisomerase I-mediated nucleosome remodeling, impairing immune response signaling.

As the general immunosuppressive activities of TDEVs involve the polarization of TAMs toward the M2 phenotype, suppression of CD8^+^ T cells, and modulation of DC function, therapies inhibiting the secretion of TDEVs from tumor cells may be one potential strategy to restore anti-tumor immunity (221, 222). These can include targeting key regulators of vesicle biogenesis (e.g., endosomal sorting complexes required for transport (ESCRT) complex, Rab proteins), vesicle trafficking inhibitors, or inhibitors of vesicle fusion with immune cells (223, 224). Inhibition of TDEV release can potentially suppress immune suppression in the TME, enhance CD8^+^ T cell survival, and increase the efficacy of checkpoint blockade therapies (225, 226). A balanced approach—targeting immunosuppressive vesicle subsets or their cargo modulation—thus offers a more promising therapeutic option. In conclusion, while exosome-based delivery systems like exoASO-STAT6 represent novel strategies for immunomodulation, the parallel development of inhibitors of the immunosuppressive functions or secretion of TDEVs would be complementary to current therapies. Exploring these possibilities has the potential to significantly enhance the translational potential of TDEV-targeted immunotherapies.

## 7 Future directions for targeting TDEV-mediated immune reprogramming

The ability of TDEVs to reprogram immune cells into immunosuppressive states has become a key factor in how tumors evade the immune system (227). To tackle this, future therapies should consider interactions between TDEVs and immune cells, especially those that influence crucial intracellular signaling pathways such as NF-κB, JAK/STAT, TGF-β, and PI3K/Akt/mTOR. Engineered EVs can be loaded with precision therapeutics, including antisense oligonucleotides (ASOs), siRNAs, CRISPR-Cas9 systems, or small-molecule inhibitors—to disrupt immunosuppressive pathways in the TME. These modified EVs selectively inhibit molecular programs driving M2-TAMs polarization while concurrently suppressing MDSC activity. Through these targeted actions, they enhance the antitumor functions of effector T and NK cells, effectively reprogramming the immune landscape toward tumor control (228–230). Moreover, using exosomes to deliver STING agonists or TLR ligands can boost the activity of antigen-presenting cells and reinstate type I IFN signaling within the immunosuppressive TME (231). This approach to exosome-based immunomodulation could be paired with immune checkpoint inhibitors (such as anti-PD-1/PD-L1 or anti-CTLA-4) to broaden and deepen the body’s antitumor immune response. Another intriguing therapeutic strategy is to inhibit the release of TDEVs (232). Several pharmacological agents have been discovered that can disrupt the pathways involved in exosome biogenesis and secretion (233). For example, the sphingomyelinase inhibitor GW4869 effectively blocks ceramide-dependent exosome formation, while inhibitors that target Rab27a/b GTPases can interfere with vesicle trafficking and fusion processes (234, 235). Additionally, suppressing components of the ESCRT machinery—like Alix and TSG101—has shown potential in reducing exosomal output (236, 237). However, these strategies face significant challenges, particularly regarding specificity. Many of the molecular targets involved in EV production are widely expressed, which complicates the development of targeted therapies. TDEVs are increasingly being valued as multifunctional platforms for therapeutic delivery with potential applications in gene editing, regenerative strategies, immunotherapy, and cancer vaccination (227, 238). The ability of EVs to modulate immune responses has opened up new avenues for the improvement of treatment outcomes, especially in the context of tumor immune evasion. However, the efficacy of EV-based therapeutics, and TDEVs more specifically, is largely dictated by the conditions under which they are produced. Parameters such as cell origin, culture conditions, and method of isolation can notably alter the composition and role of EVs, which is the key challenge for their standardization and clinical application.

There is still no consensus on a single protocol for isolation of homogeneous and functionally uniform EV populations (239). Traditional methods like ultracentrifugation, polymer-based precipitation, and column filtration can yield large quantities of EVs but lack the specificity to enrich for subtypes carrying specific molecular cargo (240). Newer approaches—like size-exclusion chromatography, immunoaffinity capture, and microfluidics-based isolation—offer greater specificity in targeting disease-relevant EV subsets and surface markers (241). An ideal platform for isolation needs to offer consistency in cargo composition, scalability, reproducibility, and compatibility with downstream clinical use. Such technical challenges have delayed the clinical implementation of EV-based immunotherapies, including for the reversal of tumor-induced immune reprogramming. While efforts have been underway to introduce Good Manufacturing Practice (GMP)-compliant workflows for the production of EVs, issues related to guaranteeing sterility, yield consistency, and therapeutic efficiency remain (242). Maybe most disturbing is the batch-to-batch heterogeneity that undermines the reproducibility that clinical-grade applications demand. These advances are particularly crucial for the use of TDEVs in precision oncology, where immune modulation must be targeted and consistent. Despite recent progress, large-scale manufacturing of EVs, particularly from tumor sources, is a major obstacle due to vesicle heterogeneity and the fact that it is hard to isolate them in therapeutic quantities (243). Consistent, clinically relevant doses of TDEVs require improvement in isolation technology and a greater understanding of EV biology. Moving forward, cross-disciplinary collaboration will be required to overcome these bottlenecks. The intersection of nanotechnology, systems biology, and bioengineering with artificial intelligence-driven analytics may pave the way for more streamlined EV production and purification techniques. Such advances will be critical to translating TDEV-based immune interventions from bench to bedside, enabling them to fulfill their full potential in next-generation cancer immunotherapies.

In the end, while the field has advanced in determining the impact of TDEVs on immune modulation, there is still work to be done in other areas. One promising area of study is examining GPI (glycosylphosphatidylinositol)-anchored proteins found in TDEVs (244). The existence of anchoring proteins and their roles in molecules of TDEV signaling pathways is not well documented, even though they are known to aid in important cellular adhesive, signaling, and immune system processes in cancer. Future investigations need to define the GPI-anchored roles of proteins in TDEVs. Also, investigate the possibility of those proteins assisting in the reprogramming of immune cells as well as determining if such proteins could be novel therapeutic targets. Developing more insight into these mechanisms may establish ways to target TDEV immunosuppressive control networks and increase mechanisms of effective anti-tumor immunity.

## 8 Conclusion

TDEVs serve as a complex and dynamic communication network between tumors and immune cells, playing a crucial role in shaping the immune environment of the TME. These vesicles are not just passive by-products of tumor activity; they actively influence immune responses, promoting immune suppression and aiding in tumor growth and spread. TDEVs can directly hinder the activation, differentiation, and cytotoxic functions of immune effector cells, or they can indirectly modulate immune responses by boosting the activity of immunosuppressive cells. TDEVs mediate multifaceted intercellular communication through their diverse molecular payload, which includes proteins, miRNAs, lipids, and metabolites. These vesicular components actively regulate critical immune pathways, notably NF-κB, PI3K/Akt/mTOR, JAK/STAT, and TGF-β signaling cascades—to coordinate fundamental biological processes. Through these mechanisms, TDEVs modulate cellular metabolism, direct immune cell differentiation, influence blood vessel formation, and promote tumor immune escape, demonstrating their central role in shaping the TME.

Even with significant progress in understanding the roles of TDEVs, several hurdles still hinder their clinical application. One major challenge is the diversity of EV populations and the absence of universally accepted markers that can reliably differentiate TDEVs from normal EVs. Current research faces two major technical challenges in studying TDEVs. First, the absence of standardized protocols for vesicle isolation, purification, and functional characterization hinders cross-study comparisons and data reproducibility. Second, the intricate signaling networks activated by TDEVs, with their frequent pathway crosstalk and redundant functions—create significant barriers to identifying viable therapeutic targets. These limitations collectively constrain both basic research and clinical translation in the TDEV field. Most of the insights we have come from *in vitro* or mouse models, and we still need to fully validate their relevance to human cancer. Looking to the future, research should concentrate on deciphering the specific functions of TDEV cargo and understanding how they exert their immunomodulatory effects across various cancer types and stages of disease.

Further investigation into the biology, heterogeneity, and therapeutic manipulation of TDEVs enhances our knowledge of cancer immunology and makes it easier to create novel, TDEV-targeted cancer treatment approaches.
